# Construction and Properties of Donor–Acceptor Stenhouse Adducts on Gold Surfaces

**DOI:** 10.1021/acs.langmuir.0c03275

**Published:** 2021-03-01

**Authors:** Dalma Edit Nánási, Attila Kunfi, Ágnes Ábrahám, Péter J. Mayer, Judith Mihály, Gergely F. Samu, Éva Kiss, Miklós Mohai, Gábor London

**Affiliations:** † MTA TTK Lendület Functional Organic Materials Research Group, Institute of Organic Chemistry, Research Centre for Natural Sciences, Magyar tudósok körútja 2, 1117 Budapest, Hungary; ‡ Laboratory of Interfaces and Nanostructures, Eötvös Loránd University, Pázmány Péter stny. 1/A, 1117 Budapest, Hungary; § Institute of Chemistry, University of Szeged, Rerrich tér 1, 6720 Szeged, Hungary; ∥ Biological Nanochemistry Research Group, Institute of Materials and Environmental Chemistry, Research Centre for Natural Sciences, Magyar tudósok körútja 2, 1117 Budapest, Hungary; ⊥ Department of Physical Chemistry and Materials Science, Interdisciplinary Excellence Centre, University of Szeged, Rerrich Square 1, H-6720 Szeged, Hungary; # Institute of Materials and Environmental Chemistry, Research Centre for Natural Sciences, Magyar tudósok körútja 2, 1117 Budapest, Hungary

## Abstract

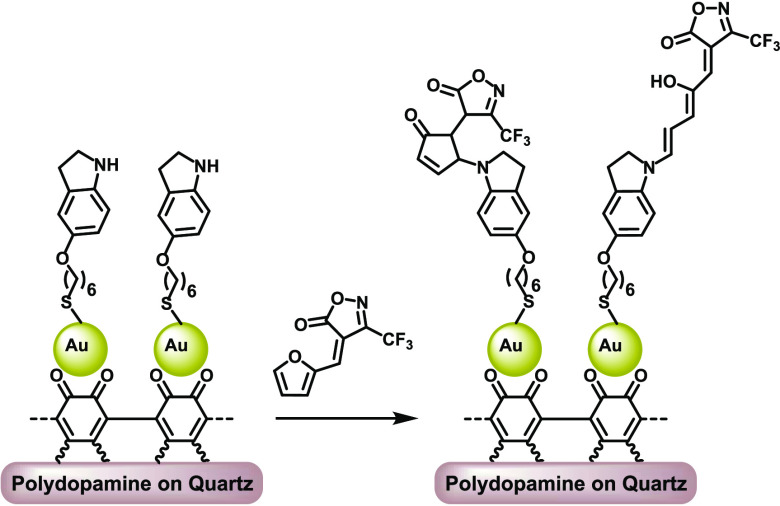

The construction of a donor–acceptor Stenhouse adduct molecular layer on a gold surface is presented. To avoid the incompatibility of the thiol surface-binding group with the donor–acceptor polyene structure of the switch, an interfacial reaction approach was followed. Poly(dopamine)-supported gold nanoparticles on quartz slides were chosen as substrates, which was expected to facilitate both the interfacial reaction and the switching process by providing favorable steric conditions due to the curved particle surface. The reaction between the surface-bound donor half and the CF_3_-isoxazolone-based acceptor half was proved to be successful by X-ray photoelectron spectroscopy (XPS). However, UV–vis measurements suggested that a closed, cyclopentenone-containing structure of the switch formed on the surface irreversibly. Analysis of the wetting behavior of the surface revealed spontaneous water spreading that could be associated with conformational changes of the closed isomer.

## Introduction

Exploiting the collective and synchronized transformations of molecular switches and motors is widely considered as one of the main avenues toward their practical applications.^1−4^ To achieve this goal, such molecules have been immobilized on nanoparticles and macroscopic surfaces to reduce their uncontrolled motion in solutions and focus their induced motion on performing tasks. These integrated organic/inorganic systems have been successfully used in a wide range of applications such as guiding liquid motion,^5^ controlling surface wettability^6^ and conductance,^7^ reversible assembly of reaction nanoflasks,^8^ or artificial muscles.^9^

Within the family of molecular switches, donor–acceptor Stenhouse adducts (DASAs) are arguably the most rapidly developing systems currently.^10^ Since their description in 2014,^11^ not only were several fundamental aspects of their switching process uncovered^12−18^ but important steps were also taken toward their applications, which include controlling drug delivery,^19,20^ wetting behavior,^21,22^ or adhesion,^23^ photopatterning,^24−26^ sensing,^27−30^ and nanoreactors.^31^ Many of these are associated with confined states of the switches. Most of the approaches to attach DASAs to different interfaces involve the physisorption of the molecules or are based on purely polymeric materials. These systems, although demonstrated to be useful in coloration/decoloration-based applications, lack the order that is present in molecular layers on silicon- or gold-based materials.^32^ Creation of nonrandom assemblies of switches and motors with increased order and more precise positioning is key to their advanced applications.^1−4^ Nevertheless, the assembly of organic/inorganic covalent hybrids using DASAs is virtually unexplored. Klajn and co-workers have constructed a molecular layer of DASAs on magnetite nanoparticles that led to the irreversible bleaching of the molecules.^33^ A similar observation was made by Oms, Versace, and co-workers upon covalently attaching DASAs to polyoxometalates.^34^ Additionally, theoretical calculations on the properties of DASA/TiO_2_ hybrids have been performed, which pointed out a stronger electronic interplay between the TiO_2_ surface and the closed form of the DASA compared to the interaction between the surface and the linear form of the switch.^35^ Apart from the above experimental works, no successful construction of DASA layers on inorganic, and particularly metallic, surfaces has been reported. The key problem appears to be the incompatibility of the donor–acceptor polyene structure of DASAs with the commonly applied thiol surface-binding group.^33,36^ In the present contribution, as an approach to bypass this incompatibility, we report the construction and properties of DASA layers on gold surfaces via an interfacial reaction approach.

## Experimental Section

### General

Commercial reagents and solvents (Sigma-Aldrich, Fluorochem, VWR) were purchased as reagent grade and used without further purification. Solvents for extraction or column chromatography were of technical quality. Opti-grade quality solvents were used for spectroscopy and sample treatment.

NMR spectra were acquired on a Varian 500 NMR spectrometer, running at 500 and 126 MHz for ^1^H and ^13^C, respectively, and on a Varian 300 NMR spectrometer, running at 300 and 75 MHz for ^1^H and ^13^C, respectively.

UV–vis spectra were measured with a Jasco V-750 spectrophotometer. Data were collected from 800 to 200 nm using 1 nm data interval, 2 nm bandwidth, and 400 nm/s scan speed.

Liquid chromatography–mass spectrometry (LC–MS) analyses of several intermediates were performed on a Shimadzu LCMS-2020 system operated in electron impact ionization (EI) mode.

Exact mass measurements were performed on a high-resolution hybrid quadrupole time-of-flight mass spectrometer (Waters Select Series IMS, Waters Corp, Wilmslow, U.K.) equipped with a Z-spray electrospray ionization source.

An optical-angle measuring and contour analysis system (OCA 15+ from DataPhysics Instruments) was applied for wetting measurements that were performed in a closed, water-saturated, and thermostated chamber. For the measured data evaluation, high-precision SCA 20 software was used to analyze the drop contours and calculate the contact angle. Wetting tension was measured using a Sigma Force Tensiometer 700.

X-ray photoelectron spectra (XPS) were recorded on a Kratos XSAM 800 spectrometer operating in fixed analyzer transmission mode, using the Mg Kα_1,2_ (1253.6 eV) excitation.

Top-down scanning electron microscopy (SEM) images were captured with an APREO C SEM microscope (ThermoFisher Scientific), operating at 1 kV accelerating voltage at a 3 mm working distance.

IR measurements were performed with a Varian 2000 (Scimitar Series) Fourier transform infrared (FT-IR) spectrometer (Varian Inc.) equipped with a mercury cadmium telluride (MCT) broad-band detector and fitted with a “Golden Gate” single-reflection diamond attenuated total reflection (ATR) accessory (Specac Ltd., U.K.). The pure quartz and the formed layers on the quartz substrate were measured in situ by pressing them onto the surface of the diamond ATR crystal with a constant pressure of 50 cN·m. In total, 128 scans were collected using a spectral resolution of 4 cm^–1^. Spectral subtraction was performed by the GRAMS/AI (7.02) software package (Thermo Galactic Inc.).

Irradiation of samples was carried out with a Euromate 3.4 W white light-emitting diode (LED), and a 10 W 620–630 nm COB LED.

### Preparation of the Modified Surfaces

#### Treatment of Quartz Slides before Polymerization

Quartz slides (∼10 mm × 25 mm) were immersed in a 1:1 mixture of H_2_SO_4_ and H_2_O_2_ (piranha solution) for 15 min. (*Caution*! Piranha is extremely corrosive and should be handled carefully!) Following the treatment, the slides were washed several times with deionized water and immediately used in the next step to prevent surface contamination.

#### Polymerization of Dopamine on Quartz Slides (Q-PDA)

Tris base (148 mg, 1.22 mmol) and Tris-HCl (75 mg, 0.48 mmol) were dissolved in deionized water (150 mL) and purged with air for 30 min. Quartz slides were immersed in the buffer mixture. A solution of dopamine hydrochloride (340 mg, 1.79 mmol) in water (20 mL) was added to the buffer solution and stirred (350 rpm) at room temperature for 24 h. The modified slides were washed with deionized water and acetonitrile, sonicated in acetonitrile (opti-grade) for 30 s, and dried by gently heating with a heat gun.

#### Preparation of Q-PDA-Au

Q-PDA slides were immersed in a screw cap vial (1 slide/vial) containing a solution of AuCl_3_ × 3 H_2_O in deionized water (250 μg/mL, 10 mL) for 3 h. After gold deposition, the slides were washed with deionized water and acetonitrile (opti-grade) and then dried by gently heating with a heat gun.

#### Preparation of Q-PDA-Au-Ind

Thioalkyl-containing indoline **5** (4.8 mg, 10 μmol) was added to a screw cap vial charged with MeOH (10 mL, *c* = 1 mM) and sonicated until complete dissolution. A Q-PDA-Au slide was immersed in the solution, and the vial was kept at 40 °C for 24 h. Then the slide was washed with copious amount of acetonitrile (opti-grade) and finally dried by gently heating with a heat gun.

#### Preparation of Q-PDA-Au-DASA

Furan adduct **8** (2.3 mg, 10 μmol) was added to a screw cap vial charged with DCM (10 mL, *c* = 1 mM) and sonicated until complete dissolution. A Q-PDA-Au-Ind slide was immersed in the solution and the vial was kept at room temperature for 24 h. Then the slide was washed with a copious amount of acetonitrile (opti-grade) and finally dried by gently heating with a heat gun.

#### Preparation of Q-PDA-DASA

Furan adduct **8** (30 mg, 130 μmol) was added to a screw cap vial charged with DCM (20 mL, *c* = 6.5 mM) and sonicated until complete dissolution. A Q-PDA slide was immersed in the solution, and the vial was kept at room temperature for up to 3 h. Then the slide was washed with a copious amount of acetonitrile (opti-grade) and finally dried under nitrogen flow.

## Results and Discussion

**Figure 1:**
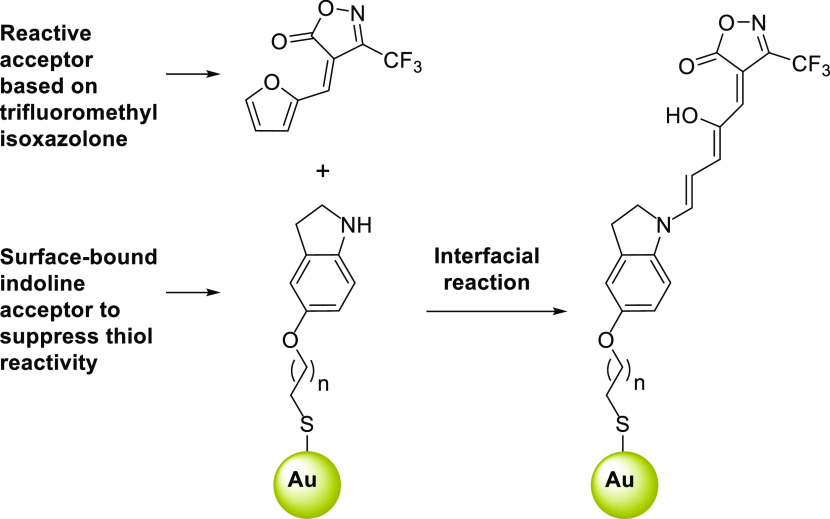
Design of DASA-on-gold via an interfacial reaction approach.

To overcome the problem of incompatibility between the thiol surface-binding group and the donor–acceptor polyene structure of DASAs, we hypothesized an interfacial reaction approach where the acceptor reacts with a surface-bound donor component (Figure 1). In this way, the thiol reactivity could be eliminated through metal binding. Regarding the molecular design for surface attachment, we turned to the recently reported third-generation DASAs, where strong carbonic acids provide the acceptor part of the switch.^37^ Among the reported carbonic acids, we chose the CF_3_-isoxazolone heterocycle as its furan adduct has exceptional reactivity with amines, which is expected to facilitate the interfacial reaction; furthermore, its CF_3_ group is an excellent reporter function in the surface analysis with XPS. Indoline was chosen as the donor moiety, which contains an alkyl thiol function for binding to gold.

**Scheme 1:**
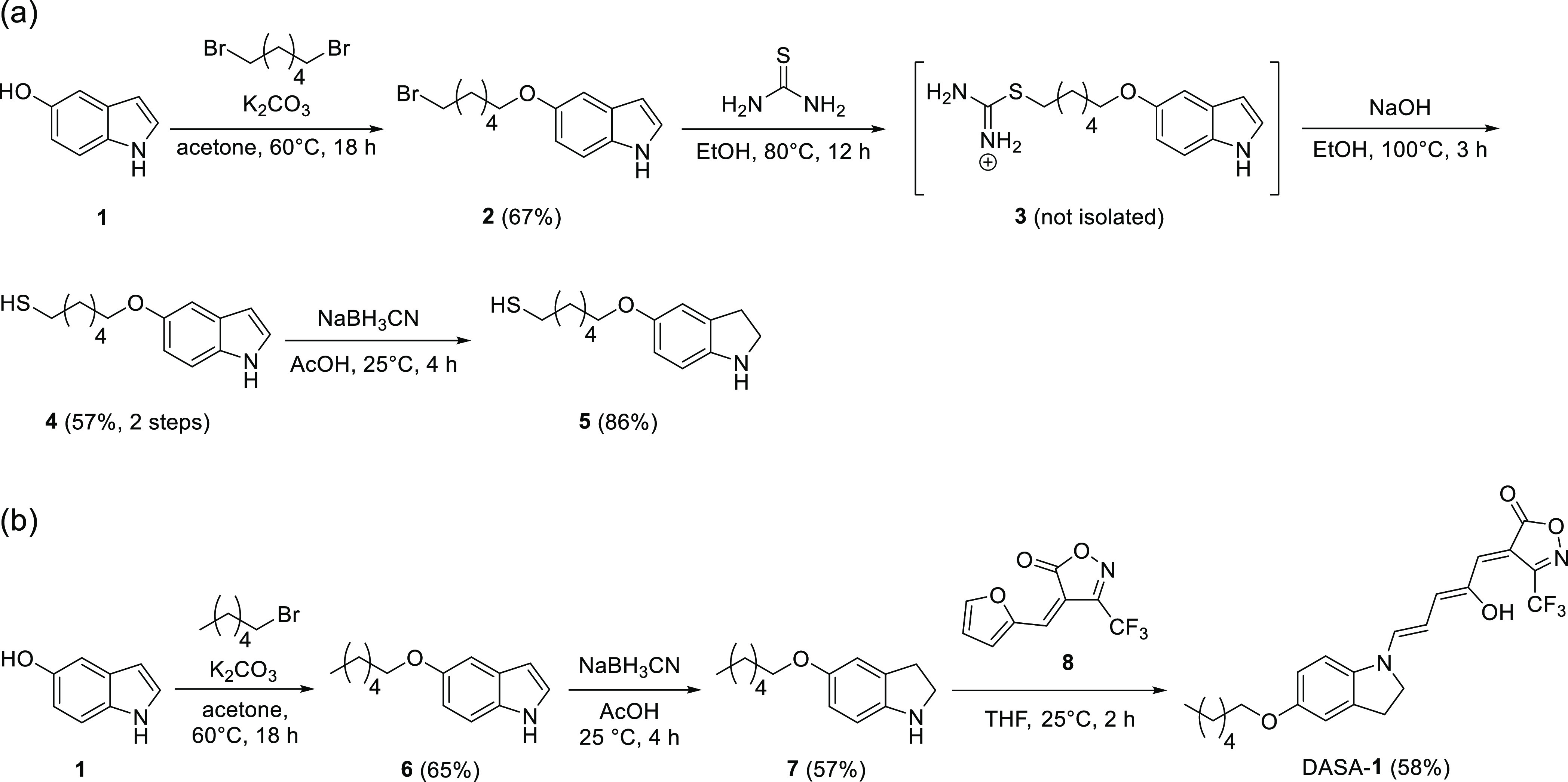
(a) Synthesis of Indoline-Thiol **5** for Gold Surface Attachment and (b) Synthesis of DASA-**1** for Solution Measurements

The synthesis of the indoline-thiol derivative **5** (Scheme 1a) was straightforward from 5-hydroxyindole **1**, which was alkylated first with 1,6-dibromohexane to obtain bromoalkyl-indol **2**. Compound **2** was transformed into the corresponding thiuronium salt **3**, which was converted to indol-thiol **4**. The desired indoline-thiol **5** was obtained by the reduction of compound **4** with NaBH_3_CN. As a solution control, DASA-**1** was also prepared through the reaction of indoline **7** with furan adduct **8** (Scheme 1b).

**Figure 2:**
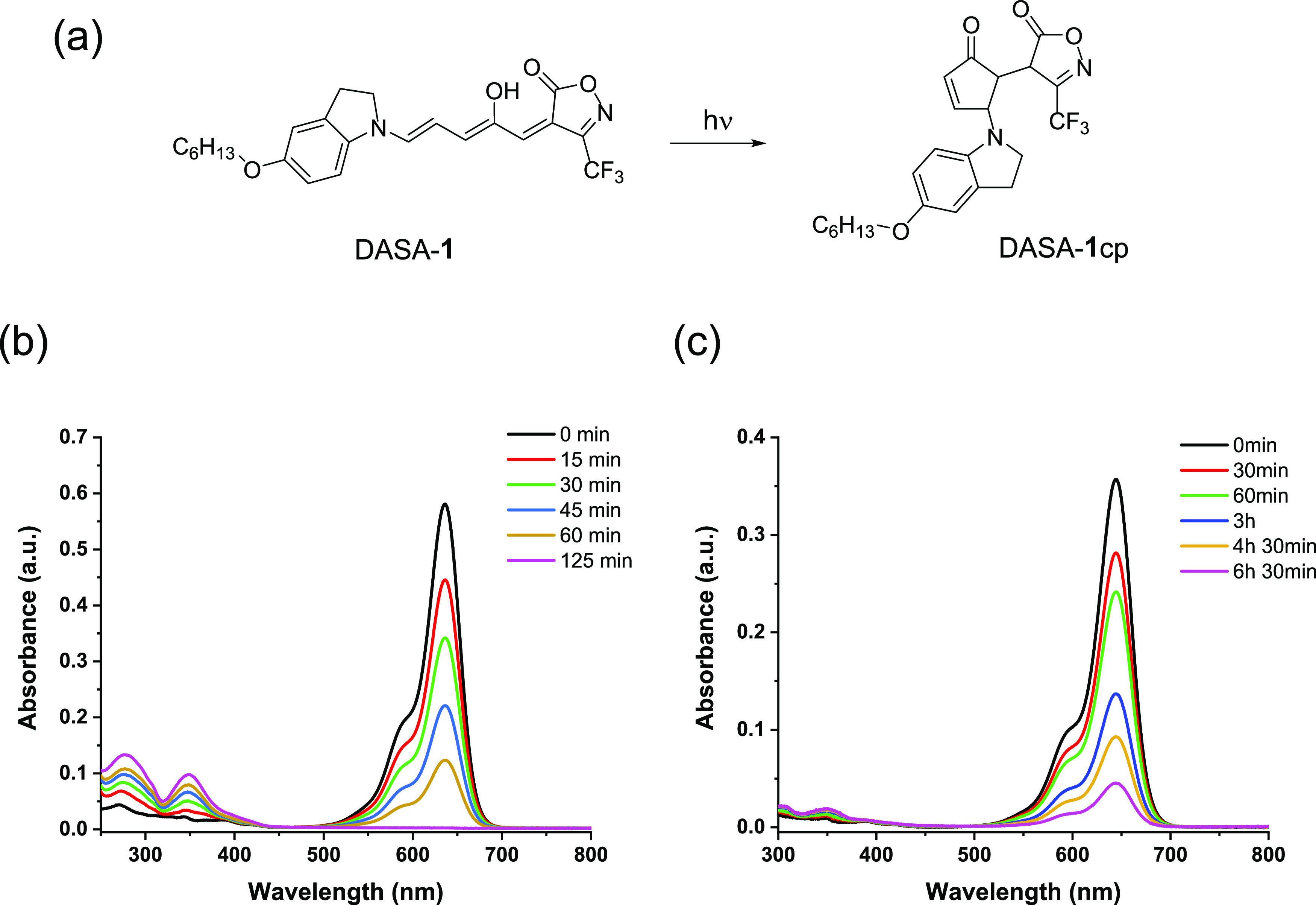
Light-induced changes in the UV–vis spectrum of DASA-**1** (a) in CHCl_3_ (b) and in toluene (c).

The solution control DASA-**1** that lacks the terminal thiol group was characterized by UV–vis spectroscopy. It showed an absorption profile that is characteristic of the triene form of the molecule (Figure 2). Upon irradiation with 620 nm light, the absorption in the visible region decreased; however, it took a rather long time to completely disappear both in toluene and CHCl_3_. Furthermore, the changes were irreversible and the triene form could not be restored (Figure S3, Supporting Information). ^1^H NMR experiments in DMSO-*d*_6_ show the disappearance of the characteristic OH and vinyl protons of the triene form of DASA-**1** upon irradiation with visible light (Figure S6, Supporting Information).

We also prepared the thiol-containing derivative of DASA-**1**; however, this derivative was found to be unstable in polar solvents^36^ and under common monolayer formation conditions,^38^ in agreement with previous reports (Section S5.2, Supporting Information). Overall, the experiments with the control molecules show that the reaction between the indoline donor and acceptor **8** occurs and leads to a stable DASA molecule, preferentially in its triene form. These findings provide a good basis for successful surface chemistry and reasonable detection of bound molecules.

**Scheme 2:**
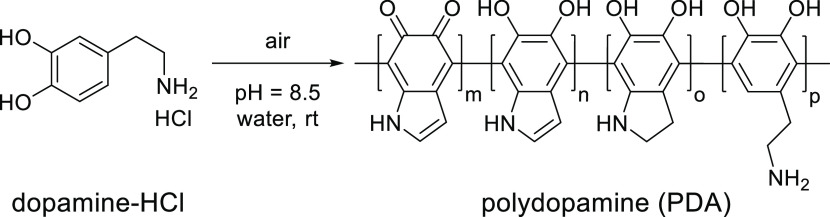
Preparation of Poly(dopamine) from Dopamine Hydrochloride

In the construction of molecular switches on surfaces via interfacial reactions, the nature of the surface is critical. Flat surfaces could lead to the formation of a dense layer of functional groups, which prevents the subsequent reaction.^38,39^ Furthermore, molecular switches need sufficient free volume for their structural changes which is hindered by high surface coverages.^40^ To avoid these difficulties associated with flat surfaces, we applied quartz slides covered with poly(dopamine) (PDA)/gold particle composite materials. We have successfully exploited this system recently in the construction of azobenzene-based dynamic interfaces.^41^ Poly(dopamine) (Scheme 2) is a mussel-inspired polymer able to adhere to virtually any surface.^42^

**Figure 3:**
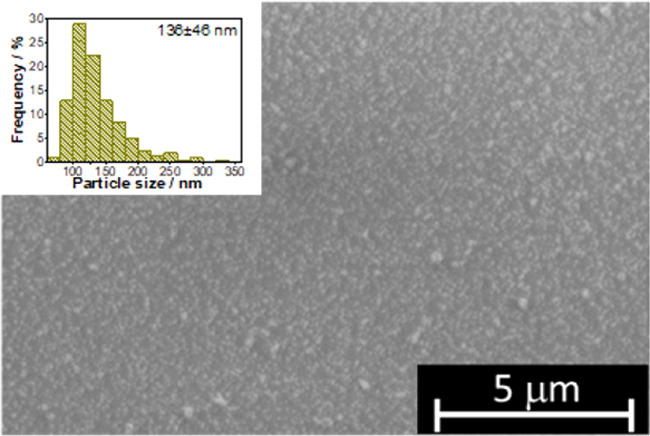
SEM image of the Q-PDA-Au surface. Inset: particle size distribution of gold particles on the surface.

**Scheme 3:**
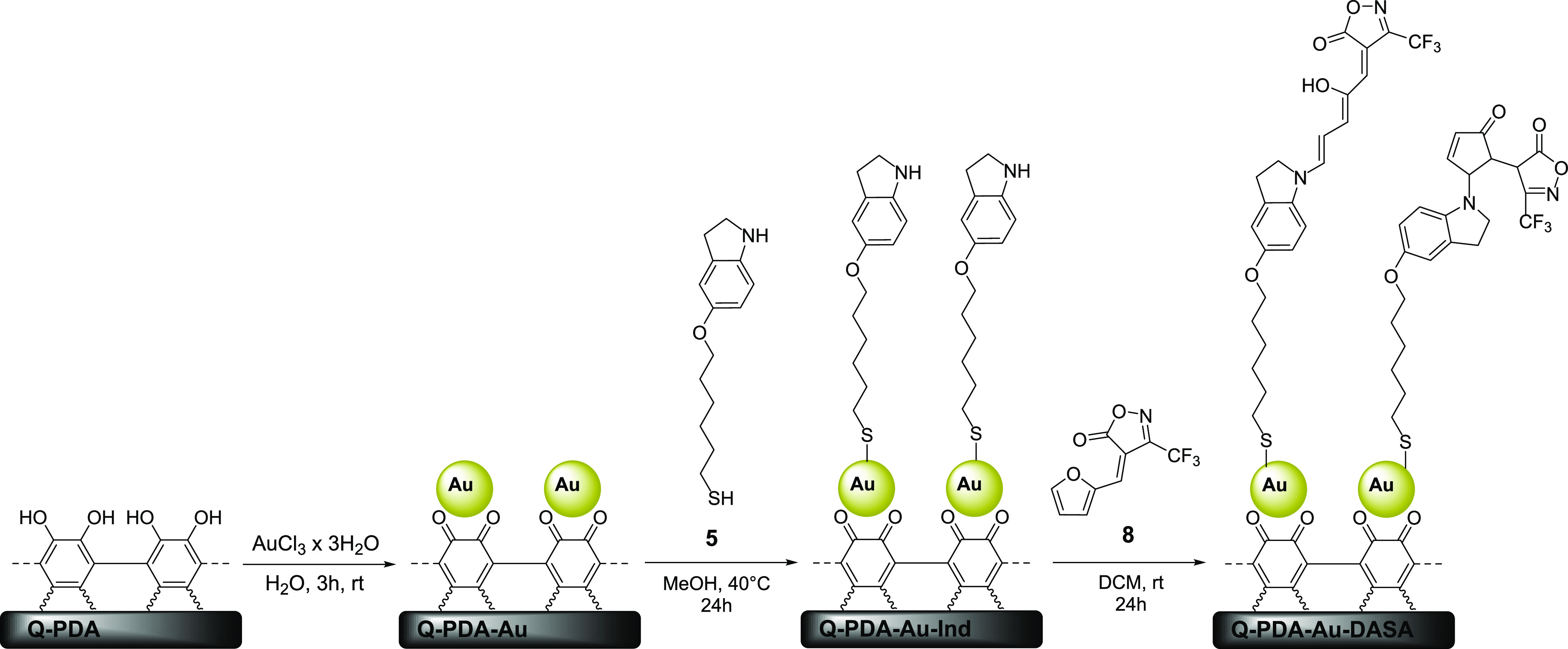
Schematic Representation of the DASA Layer Construction on a PDA/Gold Composite Surface via an Interfacial Reaction Approach

We used its monomer, dopamine hydrochloride, to cover quartz slides with the polymer under basic aqueous aerobic conditions. The redox-active catechol moieties of the PDA layer were then responsible for creating Au particles without the need for any reducing and/or capping agents, just by immersing the slide in the aqueous solution of AuCl_3_ × 3H_2_O (Scheme 3). SEM analysis of Q-PDA-Au revealed a rather uniform coverage of Q-PDA with gold particles with an average diameter of 136 nm (Figure 3). The curved Au particles were expected to facilitate both the interfacial reaction and the switching process.^43−45^

To test the feasibility of an interfacial reaction between the donor and acceptor components, we first assembled an indoline terminal molecular layer on a quartz slide that was covered with a PDA/Au composite (Q-PDA-Au-Ind) (Scheme 3).

**Figure 4:**
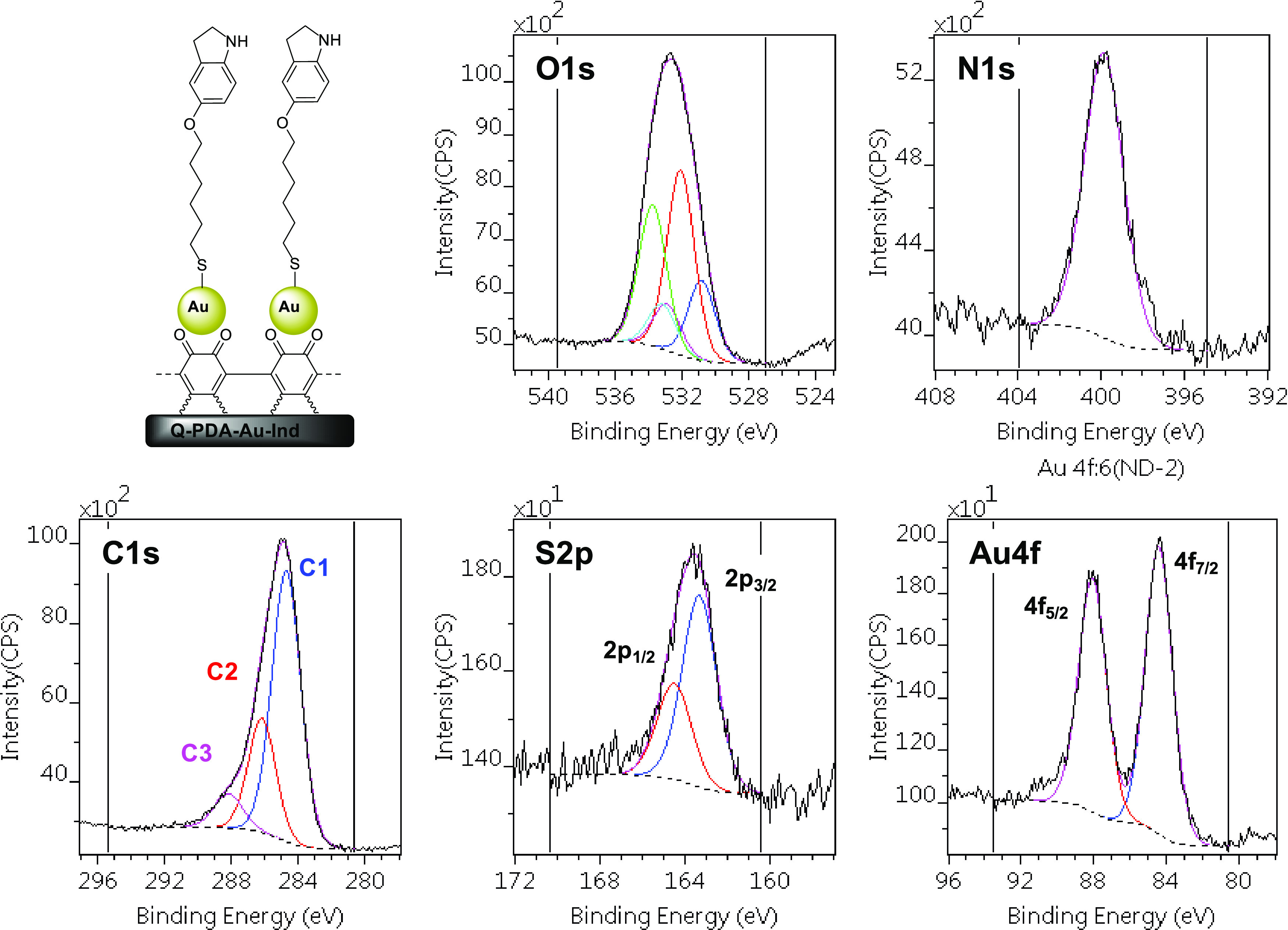
High-resolution X-ray photoelectron spectra of Q-PDA-Au-Ind.

Upon surface modification, the advancing and receding water contact angles of the relatively hydrophilic Q-PDA-Au surface (θ_adv_ = 26.3° (±2.8), θ_rec_ = 8.3° (±2.4)) were considerably increased (θ_adv_ = 73.4° (±1.9), θ_rec_ = 14.1° (±3.2)), suggesting the formation of an organic layer. Furthermore, the presence of the indoline derivative on the surface was also studied by XPS (Figure 4). Although the complexity of the PDA polymer on the surface that contains the same elements (C, O, N) as the attached molecules makes the complete resolution of the states somewhat difficult,^41^ the formation of the indoline layer is strongly supported by the XPS results. Importantly, the binding of the thiol, which is present exclusively in the indoline derivative, clearly occurred to the gold particles. The N 1s signal suggests the presence of N atoms attached to an aryl ring, which can originate partially from the PDA and partially from the indoline ring system in compound **5**. Carbon atoms including C–H and C–C (C1), C-heteroatom (C2), and C=O (C3) types are identified, among which the contribution of the C1 type is dominant (Figure 4). This indicates the presence of the hexyl chains of the indoline-thiol layer and also suggests a well-covered surface (for further details, see Section S7, Supporting Information).

**Figure 5:**
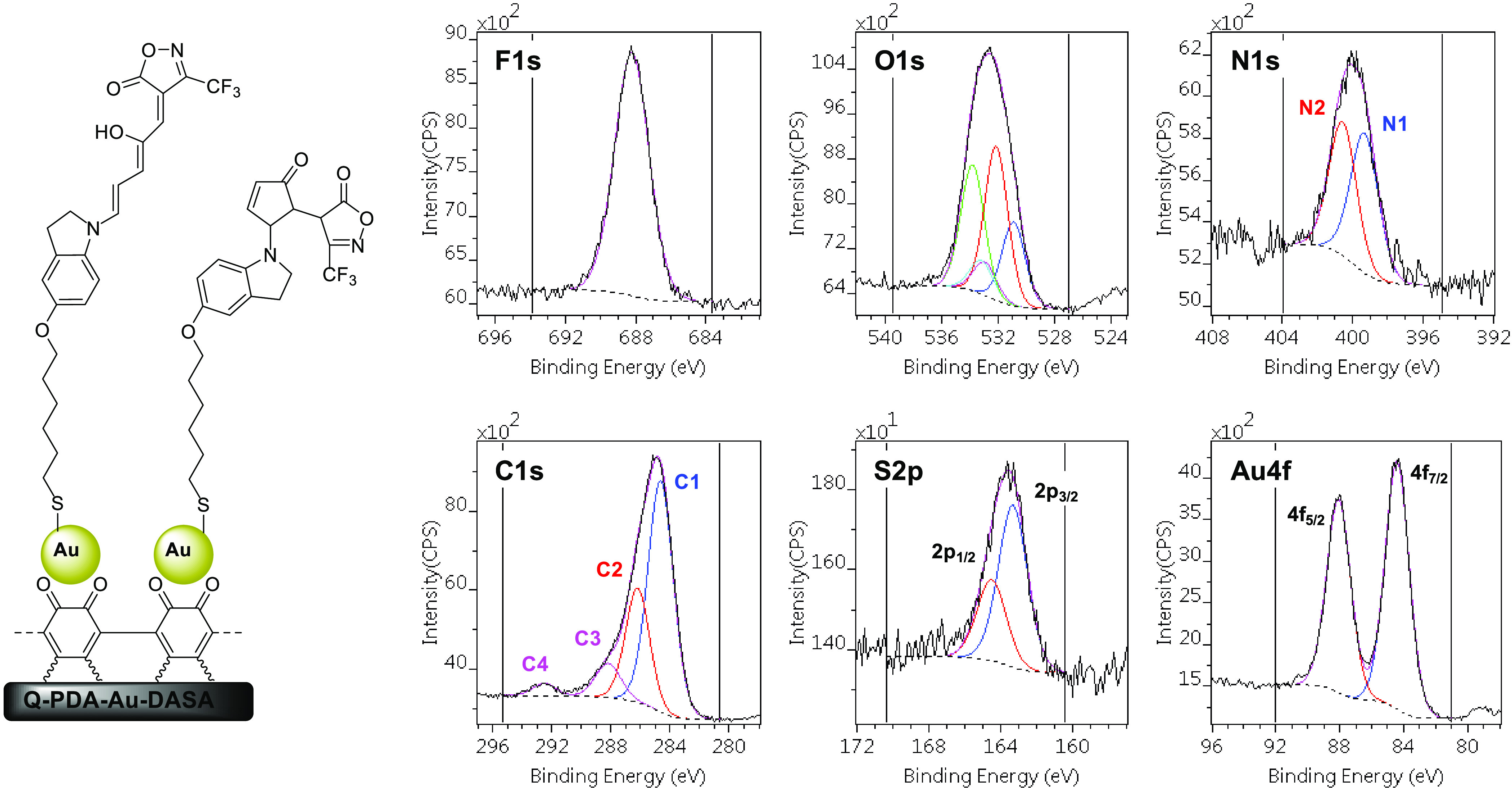
High-resolution X-ray photoelectron spectra of Q-PDA-Au-DASA.

The interfacial reaction between Q-PDA-Au-Ind and acceptor **8** was performed by immersing the slides in a DCM solution of **8** at room temperature for 24 h, which were then rinsed with a copious amount of acetonitrile and finally dried. The occurrence of the reaction was evidenced by XPS measurements (Figure 5).

The XPS results demonstrate that the heteroatoms present exclusively in the DASA molecules (S, F) are observed on the surface in well-detectable quantity. The appearance of both new types of N 1s and C 1s signals is also indicative of the DASA formation. These new N 1s and C 1s signals can be assigned as the nitrogen (O–N–C, N2) and the CF_3_ carbon (C4), respectively, of the isoxazolone ring. The composition of the various chemical states (Table S2, Supporting Information) gives more information on the surface-bound DASA molecules. The ratio of the F, CF_3_ carbon (C4 in Figure 5), and sulfur species is very close to the expected 3:1:1 value, proving that the whole molecule is present on the surface.

**Figure 6:**
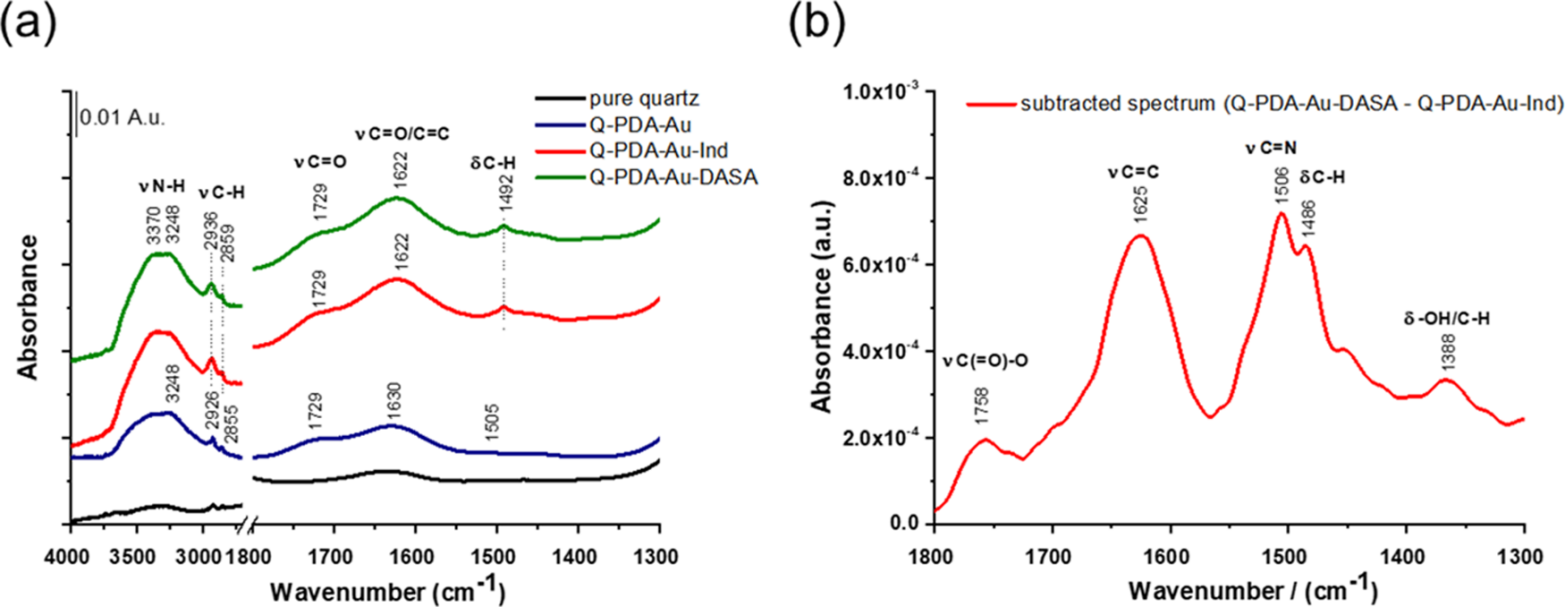
(a) ATR-IR spectra of the studied surfaces and (b) difference spectrum of Q-PDA-Au-DASA and Q-PDA-Au-Ind.

We performed an IR study using the attenuated total reflection (ATR) technique to further characterize the constructed surfaces (Figure 6). The formation of the poly(dopamine) layer could be evidenced by the presence of small, but well-identifiable, bands (Figure 6a). The bands at 2926 and 2855 cm^–1^ belong to stretching vibrations of CH_2_ moieties. The bands at 3248 cm^–1^ might belong to unreacted N–H groups. The bands at 1729 and 1630 cm^–1^ can be assigned to stretching vibrations of carbonyl groups, the latter being conjugated with C=C moieties.^46^ Upon treatment of Q-PDA-Au with indoline-thiol **5**, the intensity and shape of CH_2_ stretching bands changed and the appearance of a new band at 3370 cm^–1^ further indicated the presence of indoline N–H groups.^47^ Although no visible spectral changes could be detected after the interfacial reaction with trifluoromethyl isoxazolone-based furan adduct **8**, careful analysis performed on the subtracted spectrum (Q-PDA-Au-DASA–Q-PDA-Au-Ind) revealed some additional absorption bands related to the final DASA layer (Figure 6b). We speculated that regardless of the thinness of the new molecular layer, the C=O groups introduced by isoxazolone surface modification might give informative signals due to the high extinction coefficient of the carbonyl. Indeed, a band at 1758 cm^–1^ appeared in the subtracted spectrum. Based on the relatively high wavenumber position, it can be assigned to lactone-type groups.^46^ The broad band around 1625 cm^–1^ belongs to C=C stretching vibrations. The doublet at 1506 and 1486 cm^–1^ might refer to C=N stretching and C–H bending, respectively, of the isoxazole-type ring.^47,48^ Unfortunately, no sign of C–F vibrations could be detected (1320–1100 cm^–1^) due to the strong overlap with the Si–O stretching of the quartz substrate.

It has to be noted that based on the XPS and IR data, it is not possible to quantify the ratio of the linear and cyclic forms of the DASA on the surface; hence, we turned to solid-state UV–vis measurements. Although the transparency of Q-PDA-Au allowed for UV–vis measurements of the surface, we did not see substantial absorption in the visible region apart from the band that corresponded to the Au particles (Figure S4, Supporting Information). This suggests that the majority of the DASAs are in the nonabsorbing closed form. We could not induce the appearance of visible absorption that is associated with the presence of the linear form of the switch even with prolonged heating (up to 30 min at 80 °C). The lack of UV–vis absorption also prevented us from using this technique, in comparison with solution spectra, to estimate the surface concentration of the switch. The irreversible formation of the cyclic isomer of DASAs is in line with previous reports on organic/inorganic DASA hybrids.^33,34^ As the reversible switching of DASAs in solution strongly depends on the polarity of the media, the proximity of the polar surface might negatively influence reversibility, just as polar solvents in solution. A potential approach to address this problem could be the systematic construction of mixed monolayers of hydrophobic alkyl chains and DASAs on inorganic surfaces, where the alkyl chains could provide an apolar environment where reversible switching might be observed.

**Figure 7:**
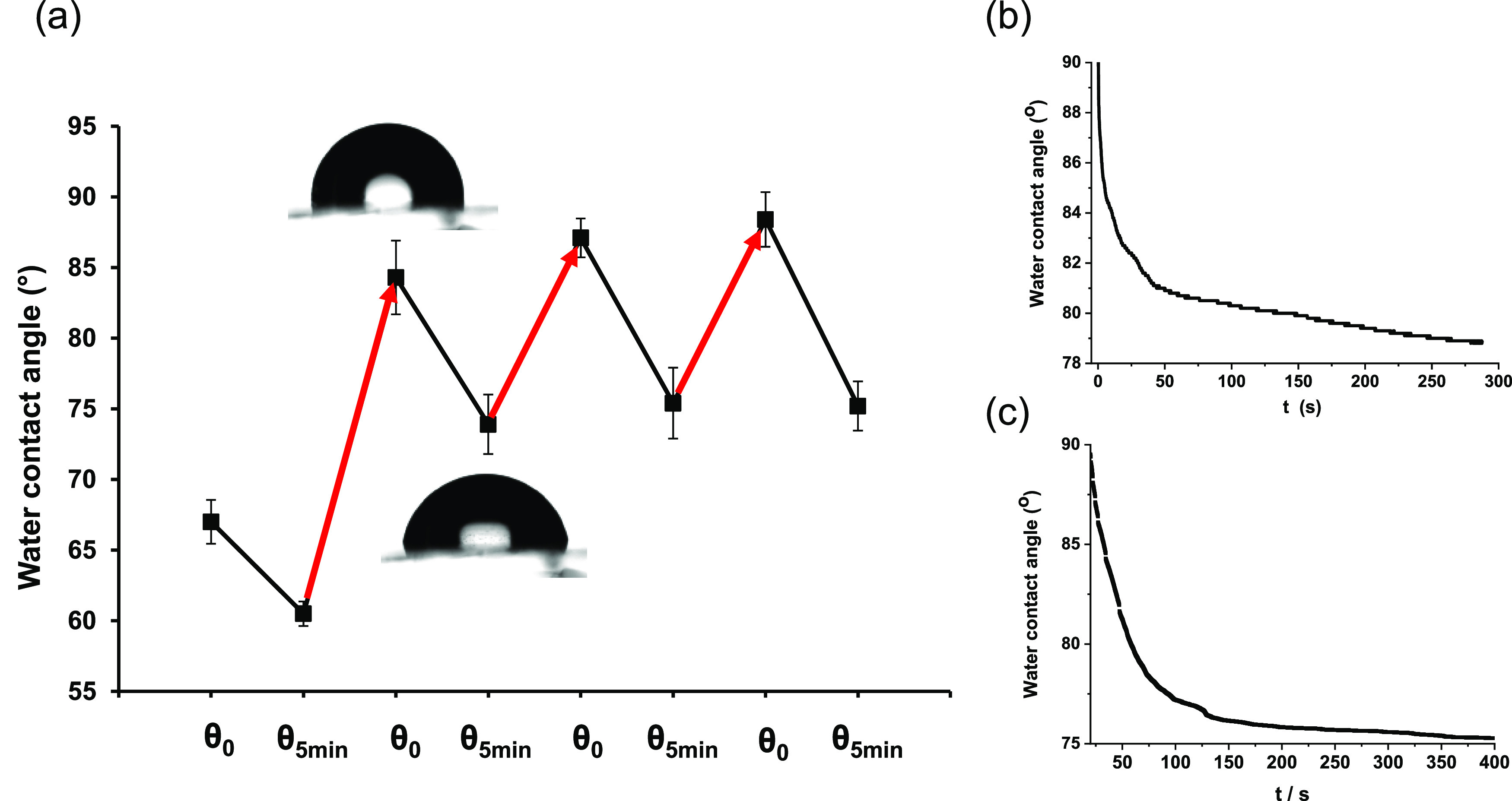
(a) Changes in water contact angles on Q-PDA-Au-DASA. Black lines correspond to the spontaneous decrease of contact angle upon droplet deposition; red lines correspond to changes after the complete immersion of the slide in water, followed by drying at 80 °C for 30 min. (b) Time dependence of the contact angle of a water droplet deposited on Q-PDA-Au-DASA. (c) Time dependence of water contact angle on Q-PDA-Au-DASA measured tensiometrically.

DASAs, apart from being light-responsive structures, have been reported to undergo water-assisted reversible isomerization as well.^14,17,49^ We tested the interaction of the Q-PDA-Au-DASA surface with water as an attempt to induce reversible isomerization of the switch at the surface. The water-induced formation of the more hydrophilic closed form—and the associated liquid spreading—could be the basis of their use in micro/nanofluidic systems. To probe potential water-induced changes, the Q-PDA-Au-DASA surface was characterized by water contact angle measurements (Figure 7). In contrast to Q-PDA, Q-PDA-Au, and Q-PDA-Au-Ind, we noticed that immediately after depositing the droplets, they started to spread on the DASA-containing surface resulting in a sharp decrease of the initial contact angle values (receding (θ_rec_) contact angles could not be measured). This phenomenon was observed on all Q-PDA-Au-DASA samples measured; however, no response to water was observed on Q-PDA-DASA, which was prepared by the direct reaction of Q-PDA^21^ and acceptor **8**. In this latter case, we could not detect the linear form of the DASA by UV–vis spectroscopy (Section S4.2, Supporting Information), although water contact angle measurements (θ_adv_ = 60.4 ± 2.4°, θ_rec_ = 9.4 ± 1.4°) and XPS (Figure S13, Supporting Information) indicated the occurrence of surface modification. Furthermore, a previously reported azobenzene-modified Q-PDA-Au surface did not show any dynamic effect upon interaction with water either.^41^ These findings ultimately suggest that the observed water droplet spreading on Q-PDA-Au-DASA is not due to the underlying PDA-Au composite. We also studied the reversibility of the process (Figure 7a). For this, water droplets were deposited on a Q-PDA-Au-DASA surface and their contact angles were measured initially (θ_0_) and after 5 min (θ_5min_). After measuring several droplets (≥6), the whole slide was immersed in water for 5 min and dried for 30 min at 80 °C. Following drying, the measurement was repeated. The reason for immersing in water was to treat the entire surface uniformly before the next measurement to avoid potential local water-induced effects. In the first round of measurements, θ_0_, on average, was around 70°, which was decreased by about 10° in 5 min. After treating the whole slide with water followed by drying, the newly measured initial contact angles were considerably increased to about 85°. This increased value could be due to the more complete elimination of water molecules that might originate initially from solvents used or water vapor in the air, from the surface at elevated temperatures. Nevertheless, the higher initial contact angle rapidly decreased by about 12°, similar to the first measurement (Figure 7b). In the following third and fourth cycles, the initial and final contact angles remained comparable with those in the second cycle (Figure 7a). Such changes in surface polarity are reminiscent of the so-called hydrophobic recovery of bulk polymeric materials, where the induced (e.g., by treatment of oxygen plasma) polar surface groups are eventually buried due to the motile polymer chains.^50,51^

The dynamics of wetting was also studied tensiometrically (Figure 7c). Wetting tension was detected on the sample plate by immersing it in water and recording the force acting along the three-phase contact line. Keeping the plate in a fixed position, a continuous increase of force and hence a decrease of contact angle was observed, which approached a steady value after approximately 5 min. This observation is in line with the spreading of water droplets deposited on Q-PDA-Au-DASA.

**Figure 8:**
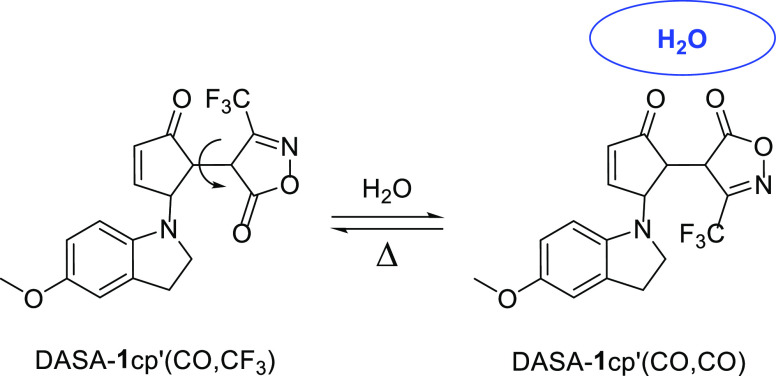
Conformational changes of DASA-**1**cp′ that could impact the wettability of Q-PDA-Au-DASA surfaces.

Importantly, upon repeated water immersion and drying cycles, the linear isomer did not form as indicated by UV–vis measurements. The apparent lack of the open form suggests that the water-induced effects are mainly not due to linear-to-cyclic isomerization cycles. We speculated that it might originate from conformational changes of DASA-**1**cp in the presence of water (Figure 8). When the surface comes in contact with water, hydrogen-bonding interactions could form with the heteroatoms present in the molecule, which would lower the surface energy. Thus, the distribution of the different surface-exposed structures would change toward the structures where H-bonds are maximized.

**Figure 9:**
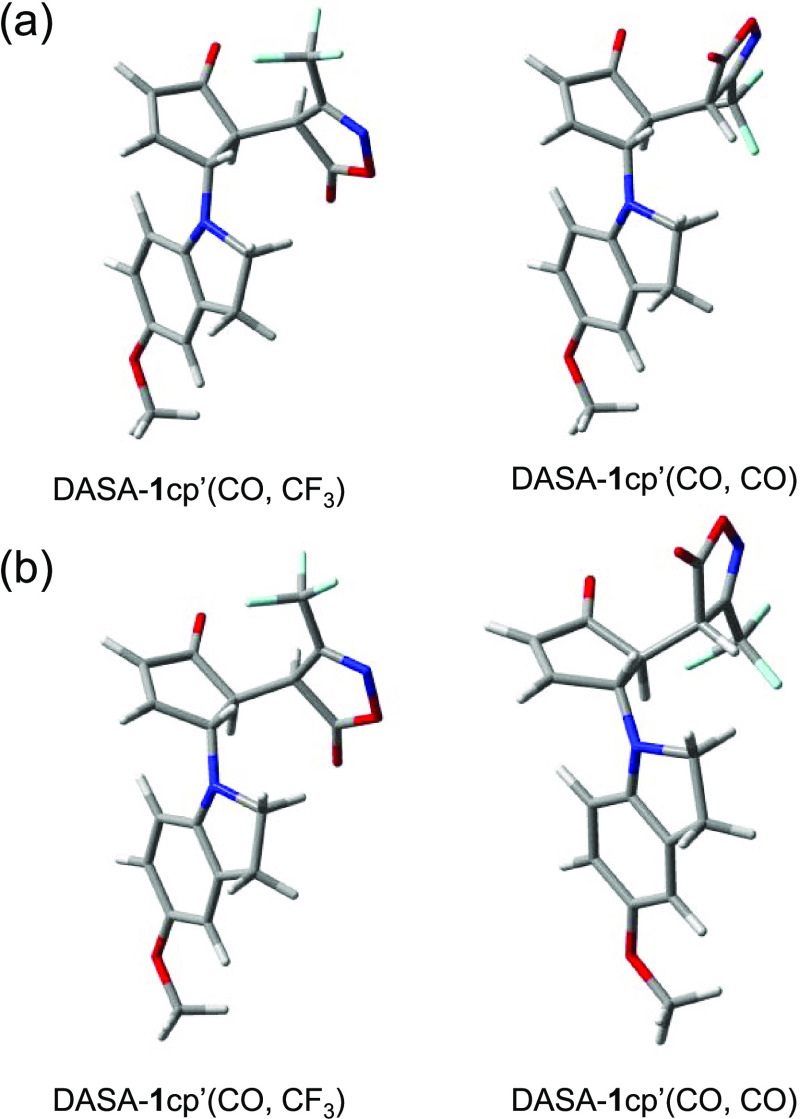
Different energy minima of DASA-**1**cp calculated in the gas phase (a) and in the water solvent model (b).

To support this possibility, we performed density functional theory (DFT) calculations^52^ on the energetics associated with the relative positions of the cyclopentenone and CF_3_-isoxazolone ring (Figure 8) at the B3LYP/6-311+g(d,p) level of theory (see also Section S8, Supporting Information). As a simplification, for calculations we considered DASA-**1**cp′ having only a methoxy group on the aryl ring. The structure where the CF_3_ group of the isoxazolone ring is oriented toward the C=O group of the cyclopentenone is denoted as DASA-**1**cp′(CO,CF_3_), while the one where the two C=O groups are in proximity is denoted as DASA-**1**cp′(CO,CO). In gas-phase calculations, the potential energy surface scan led to two energy minima that approximately corresponded to the expected functional group orientations (Figure 9a). The energy difference between the two forms was found to be around 0.2 kcal/mol, favoring DASA-**1**cp′(CO,CO) over DASA-**1**cp′(CO,CF_3_). This energy difference corresponds to a Boltzmann distribution where approximately 59% of the molecules are in the DASA-**1**cp′(CO,CO) form. Performing the calculations in water (Figure 9b), using an implicit solvent model, led to an increased energy difference of about 1 kcal/mol between the two forms that corresponds to a Boltzmann distribution of 83:17, favoring DASA-**1**cp′(CO,CO). Hence, it is not unreasonable, especially in the presence of explicit solvent–DASA interactions, that such water-induced conformational changes impact the wettability of the surface.

## Conclusions

In conclusion, we showed that the construction of a DASA layer on gold using a thiol surface-binding group is possible through an interfacial reaction approach, where the molecule is assembled on the surface from its two, donor and acceptor, halves. In this way, the reactivity of the thiol toward the conjugated structure of the switch could be suppressed. To facilitate the interfacial reaction and then the potential photochemical switching, as a surface we used gold nanoparticles supported on a poly(dopamine)-covered quartz slide. Both processes were expected to be less hindered on the curved surface of the nanoparticles. The successful construction of the molecule was confirmed by XPS analysis; however, UV–vis measurements suggested the formation of the closed cyclopentenone form of the DASA. Water contact angle measurements indicated spontaneous water spreading on the surface, which was associated with the water-induced interconversion of rotamers of the closed form having different functional group orientations. This was also supported by theoretical calculations. Although interfacial reactions might turn to be a general approach to construct DASAs on inorganic and metal surfaces, the problem of irreversible bleaching on such surfaces remains unsolved. So far, reversible switching of surface-confined DASAs has been shown only in polymeric systems.

## Supporting Information Available

Synthetic procedures and characterization data for compounds **5**, **8** and DASA-**1**; preparation of Q-PDA-Au-DASA and Q-PDA-DASA surfaces; solid state UV–vis spectrum of Q-PDA-Au-DASA; ^1^H NMR spectrum of DASA-**1**-SH in DMSO-*d*_6_; time-dependence of water contact angle on Q-PDA-Au-DASA surfaces; assignation of chemical states; the potential energy surface scans of DASA-**1**cp′ around the C–C bond (PDF)
