# Supplementary material for: Construction and Properties of Donor–Acceptor Stenhouse Adducts on Gold Surfaces
Source: Langmuir. 2021 Mar 1;37(10):3057–66. doi: 10.1021/acs.langmuir.0c03275 (PMC8031373; doi:10.1021/acs.langmuir.0c03275)
Supplement: Supplementary file 1 — la0c03275_si_001.pdf [file la0c03275_si_001.pdf]

Supporting Information

Construction and properties of donor-acceptor Stenhouse adducts on gold surfaces

Dalma Edit Nánási,[a,] Attila Kunfi,[a,] Ágnes Ábrahám,[b] Péter J. Mayer,[a,c] Judith Mihály,[d] Gergely F. Samu,[e] Éva Kiss,[b] Miklós Mohai,[f] Gábor London[a,]*

[a] MTA TTK Lendület Functional Organic Materials Research Group, Institute of Organic Chemistry, Research Centre for Natural Sciences, Magyar tudósok körútja 2., 1117 Budapest, Hungary

[b] Laboratory of Interfaces and Nanostructures, Eötvös Loránd University, Pázmány Péter stny. 1/A, 1117, Budapest, Hungary

[c] Institute of Chemistry, University of Szeged, 6720 Szeged, Rerrich tér 1., Hungary

[d] Biological Nanochemistry Research Group, Institute of Materials and Environmental Chemistry, Research Centre for Natural Sciences, Magyar tudósok körútja 2., 1117 Budapest, Hungary

[e] Department of Physical Chemistry and Materials Science, Interdisciplinary Excellence Centre, University of Szeged, Rerrich Square 1, Szeged H-6720, Hungary

[f] Institute of Materials and Environmental Chemistry, Research Centre for Natural Sciences, Magyar tudósok körútja 2., 1117, Budapest, Hungary

Email: london.gabor@ttk.hu

Table of Contents

S1 General Information S2
S2 Synthesis..S3
S3 Preparation of the modified quartz surfaces..S8
S4 UV-Vis measurements S9
S4.1 Irradiation of DASA-1 ....S9
S4.2 UV-Vis data on Q-PDA-Au-DASA and Q-PDA-DASA S11
S5 NMR-experiments..S12
S5.1 Irradiation of DASA-1 ....S12
S5.2 Stability of DASA-1-SH S13
S6 Contact angle measurements..S15
S7 XPS analyses S16
S8 Computational details..S21
S9 NMR spectra..S25

S1 General Information

Commercial reagents, solvents and catalysts (Aldrich, Fluorochem, VWR) were purchased as reagent–grade and used without further purification. Solvents for extraction or column chromatography were of technical quality. For spectroscopy and sample treatment opti-grade quality solvents were used. Organic solutions were concentrated by rotary evaporation at 25-40 °C. Thin layer chromatography was carried out on SiO_2–layered aluminium plates (60778-25EA, Fluka). Column chromatography was performed using SiO_2–60 (230–400 mesh ASTM, 0.040–0.063 mm from Merck) at 25 °C or using a Teledyne Isco CombiFlash® Rf+ automated flash chromatographer with silica gel (25-40 μm, Redisep Gold®). Room temperature refers to 25(+/- 1)°C.

NMR spectra were acquired on a Varian 500 NMR spectrometer, running at 500 and 126 MHz for ^{1}H and ^{13}C, respectively, and on a Varian 300 NMR spectrometer, running at 300 and 75 MHz for ^{1}H and ^{13}C, respectively. The residual solvent peaks were used as the internal reference. Chemical shifts (δ) are reported in ppm. The following abbreviations are used to

indicate the multiplicity in ^{1}H NMR spectra: s, singlet; d, doublet; t, triplet; q, quartet; p, pentett; m, multiplet. ^{13}C NMR spectra were acquired on a broad band decoupled mode.
UV-Vis spectra were measured with a Jasco V-750 spectrophotometer. Data were collected from 800 nm to 200 nm using 1 nm data interval, 2 nm bandwidth, and 400 nm/s scan speed.
LC-MS analyses of several intermediates were performed on a Shimadzu LCMS-2020 System operated in electron impact ionization (EI) mode.
Exact mass measurements were performed on a high-resolution hybrid quadrupole-orbitrap mass spectrometer (Waters Select Series IMS, Waters Corp., Wilmslow, U.K.) equipped with Z-spray electrospray ionization source. Samples were dissolved in acetonitrile-water 1:1 (V/V) solvent mixture containing 0.1% (V/V) formic acid. Solutions were directly introduced into the ion source using the built-in syringe pump of the instrument. Leucine enkephalin peptide was used as a reference material for mass calibration.
The optical angle measuring and contour analysis system of the OCA 15+ from Dataphysics was applied for wetting measurements that were performed in a closed, water-saturated and thermostated chamber. For the measured data evaluation, the high-precise SCA 20 software was used to analyze the drop contours and calculate the contact angle. Wetting tension was measured using a Sigma Force Tensiometer 700.
X-ray photoelectron (XPS) spectra were recorded on a Kratos XSAM 800 spectrometer operating in fixed analyzer transmission mode, using Mg K$\alpha_{1,2}$ (1253.6 eV) excitation.
Irradiation of samples were carried out with a Spectroline ENB-280C/FE 8W 365 nm UV light source, a Euromate 3.4W white LED, and a 10W 620-630 nm COB LED.

S2 Synthesis

Synthesis of indoline **5**

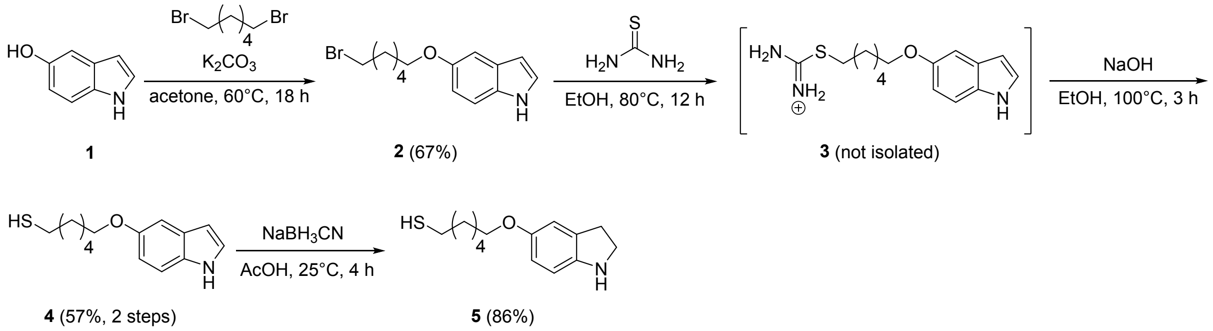

5-((6-bromohexyl)oxy)-1H-indole (2) 5-Hydroxyindole (**1**) (2.00 g, 15.02 mmol), 1,6-dibromohexane (18.32 g, 75.10 mmol) and K_2CO_3 (6.23 g, 45.06 mmol) were mixed in acetone (150 mL). The reaction mixture was refluxed under N_2 for 18 h. The progress of the reaction was followed by TLC (SiO_2, *n*-hexane / EtOAc, 5/1). Upon completion, the product mixture was allowed to cool to room temperature, filtered through a pad of Celite, and the solvent was removed by rotary evaporation. The crude product was further purified by column chromatography (*n*-hexane → *n*-hexane / EtOAc (30%)). Product **2** was obtained as a white solid (2.99 g, 67%).

^{1}H NMR (500 MHz, $CDCl_3$) δ = 8.02 (s, 1H), 7.27 (d, *J* = 8.9 Hz, 1H), 7.17 (t, *J* = 2.8 Hz, 1H), 7.12 (d, *J* = 2.2 Hz, 1H), 6.87 (dd, *J* = 8.8, 2.4 Hz, 1H), 6.48 (t, *J* = 2.0 Hz, 1H), 4.02 (t, *J* = 6.4 Hz, 2H), 3.43 (t, *J* = 6.8 Hz, 2H), 1.96 – 1.76 (m, 4H), 1.56 – 1.49 (m, 4H) ppm; ^{13}C NMR (126 MHz, $CDCl_3$) δ = 153.58, 131.01, 128.33, 124.79, 112.91, 111.60, 103.61, 102.37, 68.61, 33.82, 32.74, 29.30, 27.98, 25.39 ppm; HRMS (ESI) *m/z:* $[M+H]^+$ Calcd for $C_{14}H_{20}BrNO^+$: 296.06445; Found 296.06447.

6-((1H-indol-5-yl)oxy)hexane-1-thiol (4) Indole **2** (500 mg, 1.69 mmol) and thiourea (270 mg, 3.55 mmol) were dissolved in EtOH (60 mL). The reaction mixture was refluxed under N_2 for 12 h. The progress of the reaction was followed by TLC (SiO_2, *n*-hexane / EtOAc, 5/1). Upon completion, the product mixture was allowed to cool to room temperature, most of the EtOH was removed by rotary evaporation and *n*-pentane (10 mL) was added upon which the thiuronium salt (**3**) was precipitated. The mixture was then immersed in an ultrasonic bath for 1 min, then the precipitate was filtered, washed with an additional amount of *n*-pentane (10 mL) and dried in air. For the hydrolysis of the salt, NaOH (142 mg, 3.55 mmol) was dissolved in a minimum amount of water and was added to the suspension of the thiuronium salt in EtOH (15 mL), which was previously purged with N_2, then refluxed for 3 h. The progress of the hydrolysis was followed by TLC (SiO_2, *n*-hexane / EtOAc, 3/1). After the reaction was complete, solvent was evaporated by rotary evaporation. Following the addition of water (50 mL), the aqueous mixture was extracted with EtOAc (2x50 mL) and the combined organic phase was dried over $MgSO_4$, filtered, and the solvent was evaporated under reduced pressure. The crude product was further purified by column chromatography (*n*-hexane → *n*-hexane / EtOAc (40%)). Product **4** was obtained as a white solid (239 mg, 57%). ^{1}H NMR (500 MHz, $CDCl_3$) δ = 8.00 (s, 1H), 7.25 – 7.23 (m, 1H), 7.15 (t, *J* = 2.8 Hz, 1H), 7.08 (d, *J* = 2.2 Hz, 1H), 6.84 (dd, *J* = 8.8, 2.4 Hz, 1H), 6.45 (t, *J* = 2.1 Hz, 1H), 3.98 (t, *J* = 6.5 Hz, 2H), 2.59 – 2.42 (m, 2H), 1.84 – 1.74 (m, 2H), 1.69 – 1.58 (m, 2H), 1.47 (m, 3H), 1.32 (t, *J* = 7.7

Hz, 1H), 1.24 (t, J = 7.4 Hz, 1H) ppm; ^{13}C NMR (126 MHz, $CDCl_3$) δ = 153.80, 131.16, 128.50, 124.91, 113.12, 111.73, 103.76, 102.57, 68.83, 34.12, 29.53, 28.31, 25.82, 24.72 ppm; HRMS (ESI) *m/z:* $[M+H]^+$ Calcd for $C_{14}H_{20}NOS^+$: 250.12601; Found 250.12592.

6-(indolin-5-yloxy)hexane-1-thiol (5) Indol **4** (200 mg; 800 µmol) was dissolved in anhydrous acetic acid (20 mL) and $NaBH_3CN$ (252 mg, 4 mmol) was added slowly. The reaction mixture was stirred under N_2 at 25°C for 4 h. The solvent was evaporated, and the crude solid was dissolved in EtOAc (20 mL) and washed with water (2x20 mL). The organic phase was dried over $MgSO_4$ and filtered. From our experience, the product has limited stability on silica, so it could not be purified by column chromatography and the reaction could not be followed by TLC. To isolate sufficiently pure product, the crude product was filtered through a pad of Celite in EtOAc, and the solvent was evaporated under reduced pressure. Product **5** was obtained as an off-white solid (174 mg**,** 86 %). ^{1}H NMR (500 MHz, CD_2Cl_2) δ = 6.72 (s, 1H), 6.58 – 6.48 (m, 2H), 3.86 (t, J = 6.5 Hz, 2H), 3.50 (t, J = 8.3 Hz, 2H), 2.97 (m, 3H), 2.57 – 2.48 (m, 2H), 1.78 – 1.70 (m, 2H), 1.68 – 1.57 (m, 2H), 1.50 – 1.42 (m, 4H), 1.37 (d, J = 5.1 Hz, 1H) ppm; ^{13}C NMR (126 MHz, CD_2Cl_2) δ = 153.43, 146.18, 131.64, 113.58, 112.87, 110.25, 69.40, 48.30, 34.64, 30.99, 29.98, 28.73, 26.16, 25.06 ppm; HRMS (ESI) *m/z:* $[M+H]^+$ Calcd for $C_{28}H_{41}N_2O_2S_2^+$: 501.26040; Found 501.26017 (the S–S dimer detected).

Synthesis of furan adduct **8**[1]

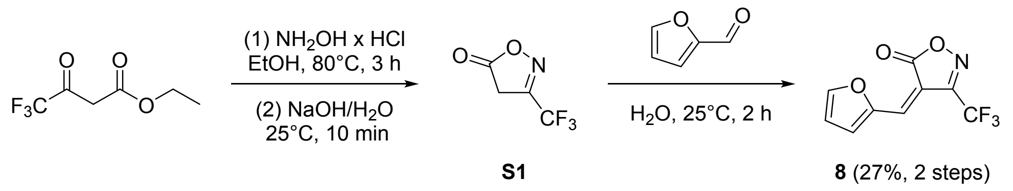

Ethyl 4,4,4-trifluoroacetoacetate (1 g, 7.6 mmol) and hydroxylamine hydrochloride (800 mg, 11.5 mmol) were refluxed in ethanol (40 mL) for 3 h. The reaction was allowed to cool to rt and the solvent was removed under reduced pressure. To the residue an aqueous solution of NaOH (4 M, 10 mL) was added, and the mixture was stirred for 10 min. The mixture was then acidified with 1M HCl, and then the aqueous phase was extracted with DCM and concentrated *in vacuo*. The resulting oil (**S1**) was dissolved in DCM (15 mL), followed by the addition of furfural (1 g, 10.4 mmol). This solution was stirred for 1 h. Then the mixture was

concentrated under reduced pressure followed by the addition of water (70 mL). The formed precipitate was filtered and washed with water (50 mL). Following filtration, product **8** was obtained as a greenish-brown solid (428 mg, 27 %).

^{1}H NMR (500 MHz, CD_2Cl_2) δ = 8.71 (d, J = 3.8 Hz, 1H), 7.98 (d, J = 1.3 Hz, 1H), 7.75 (s, 1H), 6.88 (dd, J = 3.9, 0.9 Hz, 1H) ppm; ^{13}C NMR (126 MHz, CD_2Cl_2) δ = 167.59, 154.42 (d, J = 37.4 Hz), 153.04, 151.03, 135.09, 130.20, 130.10, 119.38 (q, J = 273.4 Hz), 107.08. ppm.

Synthesis of DASA-**1**

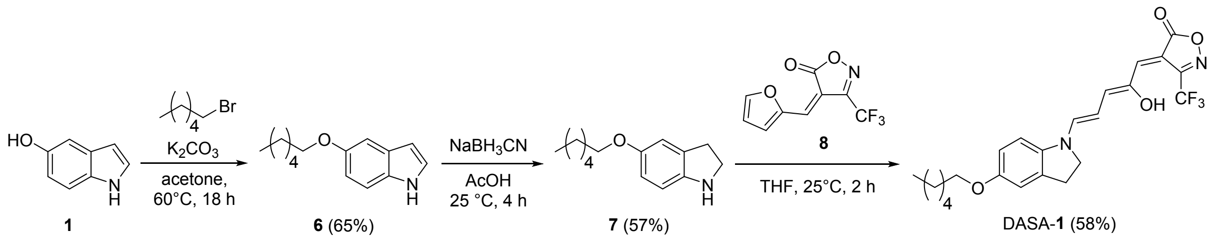

5-(hexyloxy)-1H-indole (6) 5-Hydroxyindole (**1**) (500 mg, 3.76 mmol), 1-bromohexane (1.86 g, 11.27 mmol) and K_2CO_3 (1.56 g, 11.27 mmol) were added to acetone (50 mL). The reaction mixture was refluxed under N_2 for 18 h. The progress of the reaction was followed by TLC (SiO_2, *n*-hexane / EtOAc, 5/1). The product mixture was allowed to cool to room temperature, filtered through a pad of Celite, and then the solvent was removed by rotary evaporation. The crude product was purified by column chromatography (*n*-hexane → *n*-hexane / EtOAc 30%). Product **6** was obtained as a white solid (528 mg, 65%).

^{1}H NMR (500 MHz, $CDCl_3$) δ = 8.02 (s, 1H), 7.26 (d, J = 8.8 Hz, 1H), 7.18 – 7.10 (m, 2H), 6.90 (dd, J = 8.8, 2.3 Hz, 1H), 6.49 (t, J = 2.1 Hz, 1H), 4.03 (t, J = 6.6 Hz, 2H), 1.88 – 1.79 (m, 2H), 1.56 – 1.46 (m, 2H), 1.38 (m, 4H), 0.95 (t, J = 7.0 Hz, 3H) ppm; ^{13}C NMR (126 MHz, $CDCl_3$) δ = 153.81, 131.13, 128.47, 124.91, 113.10, 111.73, 103.75, 102.46, 69.08, 31.80, 29.62, 25.96, 22.77, 14.18 ppm; HRMS (ESI) *m/z:* $[M+H]^+$ Calcd for $C_{14}H_{20}NO^+$: 218.15394; Found 218,15389.

5-(hexyloxy)indoline (7) Compound **6** (528 mg, 2.43 mmol) was dissolved in anhydrous acetic acid (30 mL) and $NaBH_3CN$ (763 mg, 12.14 mmol) was added to the solution in portions. When the addition of the solid was complete, the reaction was stirred under N_2 at 25 °C for 4 h. The progress of the reaction was followed by TLC (SiO_2, *n*-hexane / EtOAc, 3/1, 1% TEA). When the reaction was complete, acetic acid was evaporated, the crude product

was dissolved in ethyl acetate (40 mL) and washed with deionised water (2 x 40 mL) and brine (30 mL). The organic phase was dried over $MgSO_4$, filtered and the solvent was evaporated by rotary evaporation. The crude product was further purified by column chromatography (*n*-hexane / TEA (1%) → *n*-hexane / EtOAc (50%) / TEA (1%)). Product **7** was obtained as a off-white solid (302 mg, 57%).

^{1}H NMR (500 MHz, CD_2Cl_2) δ = 6.72 (d, J = 0.8 Hz, 1H), 6.58 – 6.49 (m, 2H), 3.86 (t, J = 6.6 Hz, 2H), 3.50 (m, 3H), 2.97 (t, J = 8.3 Hz, 2H), 1.80 – 1.64 (m, 2H), 1.48 – 1.39 (m, 2H), 1.37 – 1.27 (m, 4H), 0.91 (t, J = 6.7 Hz, 3H) ppm; ^{13}C NMR (126 MHz, CD_2Cl_2) δ = 153.63, 146.04, 131.67, 113.59, 112.87, 110.32, 69.58, 48.31, 32.26, 31.00, 30.09, 26.36, 23.24, 14.40 ppm; HRMS (ESI) *m/z:* $[M+H]^+$ Calcd for $C_{14}H_{22}NO^+$: 220.16959; Found 220.16952.

DASA-1 Furan adduct **8** (53 mg, 230 µmol) and indoline **7** (50 mg, 230 µmol,) were separately dissolved in THF (1-1 mL), mixed, then diluted with an additional amount of THF (2 mL). The solution was stirred at 25°C for 1 h then cooled to 0°C and stirred for additional 1 h. Upon stirring, the initially light-colored solution of the starting materials turned to bright purple-blue. During the course of the reaction, the product DASA-**1** precipitated. Ice-cold diethyl ether (4 mL) was added to the mixture and the dark blue, solid product was filtered and the residue washed with ice-cold diethyl ether, and dried. The product DASA-**1** was obtained as a vivid blue solid (60 mg, 58 %).

^{1}H NMR (500 MHz, DMSO-d_6) δ = 11.74 (s, 1H), 8.81 (d, J = 11.3 Hz, 1H), 7.66 (d, J = 8.9 Hz, 1H), 7.31 (d, J = 13.4 Hz, 1H), 7.12 – 6.97 (m, 2H), 6.48 (t, J = 12.3 Hz, 1H), 5.99 (s, 1H), 4.47 (t, J = 6.6 Hz, 2H), 4.02 (t, J = 6.4 Hz, 2H), 3.30 (t, J = 6.9 Hz, 2H), 1.71 (dd, J = 13.9, 6.7 Hz, 2H), 1.50 – 1.19 (m, 6H), 0.88 (s, 3H) ppm; ^{13}C NMR (126 MHz, DMSO-d_6) δ = 176.14, 160.29, 152.28, 151.74, 148.37, 138.56, 134.25, 121.70, 117.76, 115.85, 115.04, 112.96, 111.98, 111.70, 87.99, 68.70, 51.55, 31.39, 28.95, 27.81, 25.55, 22.50, 14.34 ppm; HRMS (ESI) *m/z:* $[M+H]^+$ Calcd for $C_{23}H_{26}F_3N_2O_4^+$: 451.18392; Found 451.18420.

S3 Preparation of the modified quartz surfaces

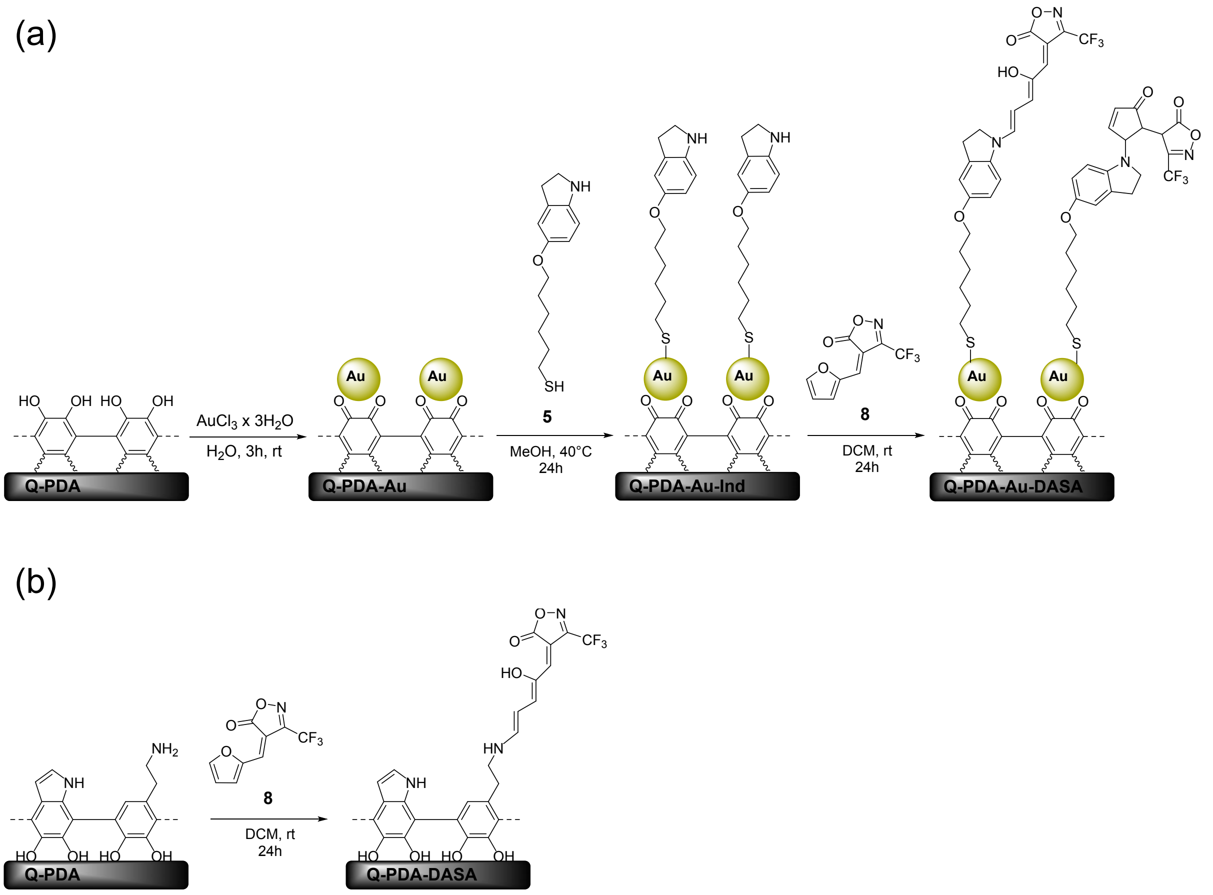

Figure S1. (a) Preparation of Q-PDA-Au-DASA and (b) Q-PDA-DASA surfaces.

Treatment of quartz slides before polymerization

Quartz slides (~ 10 mm x 25 mm) were immersed in a 1/1 mixture of H_2SO_4 and H_2O_2 (*piranha solution*) for 15 min. (*Caution! Piranha is extremely corrosive. Operate carefully!*) Following the treatment, the slides were washed several times with deionized water and immediately used in the next step to prevent surface contamination.

Polymerization of dopamine on quartz slides (Q-PDA)

Tris base (148 mg, 1.22 mmol) and Tris-HCl (75 mg, 0.48 mmol) were dissolved in deionized water (150 mL) and purged with air for 30 min. Quartz slides were immersed into the buffer mixture. A solution of dopamine hydrochloride (340 mg, 1.79 mmol) in water (20 mL) was added to the buffer solution and stirred (350 rpm) at room temperature for 24 h. The modified slides were washed with deionized water and acetonitrile, then sonicated in acetonitrile (opti-grade) for 30 sec and dried by gently heating with a heat gun.

Preparation of Q-PDA-Au

Q-PDA slides were immersed in a screw cap vial (1 slide/vial) containing a solution of $AuCl_3 \times 3\ H_2O$ in deionized water (250 µg/mL, 10 mL) for 3 h. After gold deposition, the slides were washed with deionized water and acetonitrile (opti-grade), then dried by gently heating with a heat gun.

Preparation of Q-PDA-Au-Ind

Thioalkyl containing indoline **S7** (4.8 mg, 10 µmol) was added to a screw cap vial charged with MeOH (10 mL, c = 1 mM) and sonicated until complete dissolution. A Q-PDA-Au slide was immersed in the solution and the vial was kept at 40°C for 24 h. Then the slide was washed with copious amount of acetonitrile (opti-grade), then dried by gently heating with a heat gun.

Preparation of Q-PDA-Au-DASA

Furan adduct **1** (2.3 mg, 10 µmol) was added to a screw cap vial charged with DCM (10 mL, c = 1mM) and sonicated until complete dissolution. A Q-PDA-Au-Ind slide was immersed in the solution and the vial was kept at room temperature for 24 h. Then the slide was washed with copious amount of acetonitrile (opti-grade), then dried by gently heating with a heat gun.

Preparation of Q-PDA-DASA

Furan adduct **1** (30 mg, 130 µmol) was added to a screw cap vial charged with DCM (20 mL, c = 6.5 mM) and sonicated until complete dissolution. A Q-PDA slide was immersed in the solution and the vial was kept at room temperature for up to 3 h. Then the slide was washed copious amount of acetonitrile (opti-grade), then dried under nitrogen flow.

S4 UV-Vis measurements

S4.1 Irradiation of DASA-1

Irradiation of the samples were carried out with a 10W, 620–630 nm COB LED source. Hellma Analytics High Precision quartz cuvettes were used for measurements with optical path length of 1.0 cm.

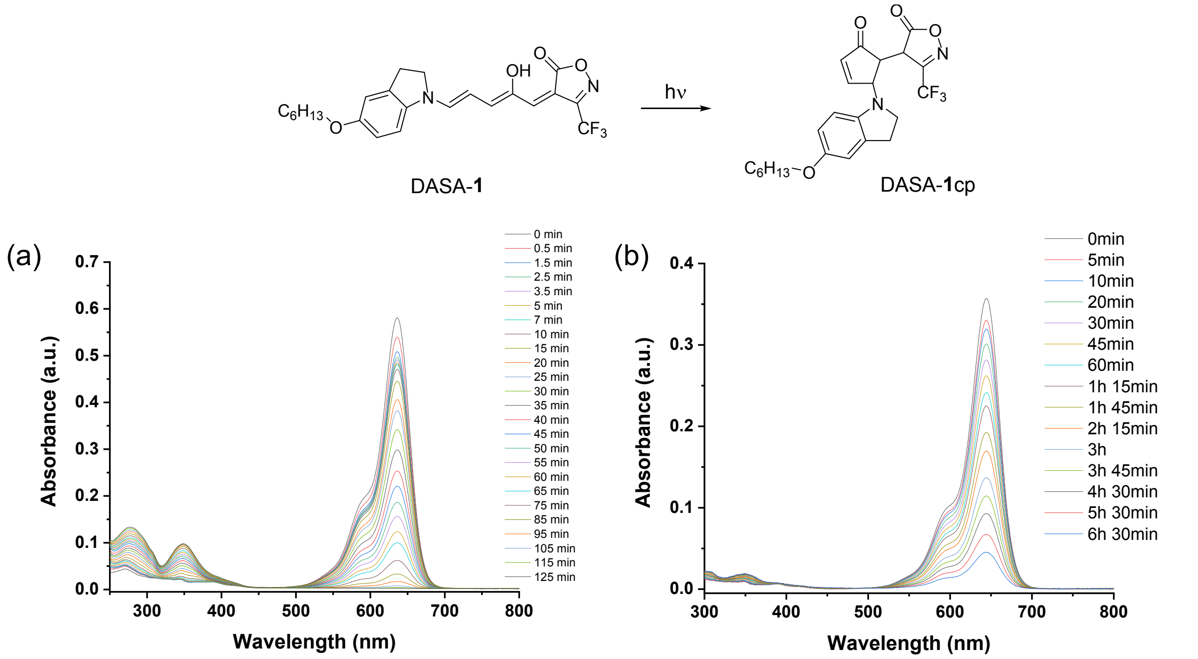

Figure S2. Conversion of DASA-**1** into DASA-**1**cp upon irradiation with 620-630 nm LED in (a) $CHCl_3$ and (b) toluene solution.

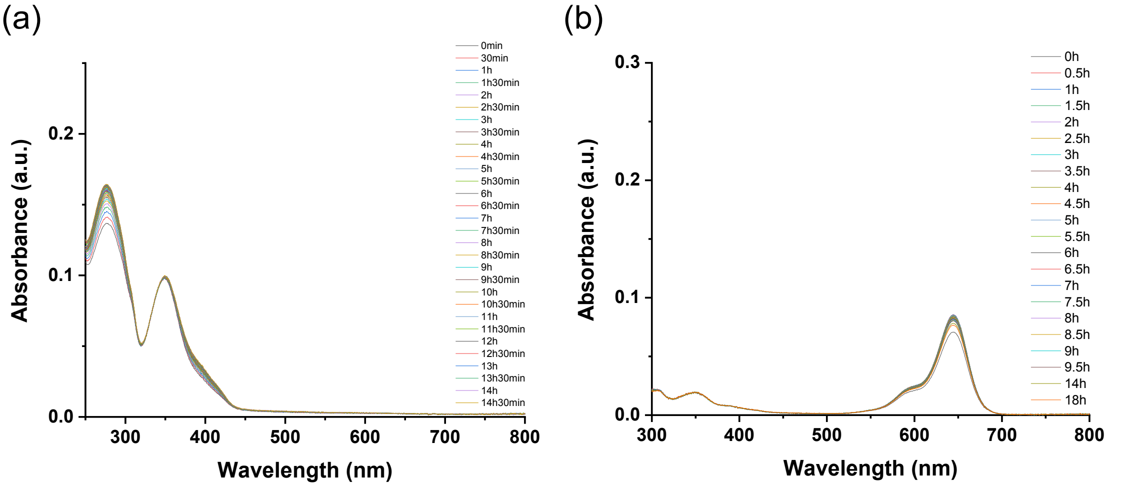

Figure S3. Monitoring thermal relaxation of DASA-**1**cp in (a) $CHCl_3$ and (b) toluene solution in the dark after irradiation of DASA-**1** with 620-630 nm LED.

S4.2 UV-Vis data on Q-PDA-Au-DASA and Q-PDA-DASA

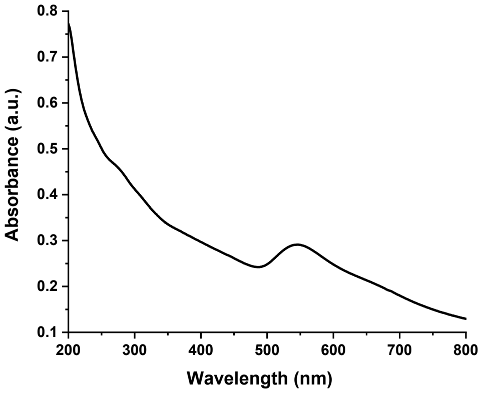

Figure S4. Solid state UV-Vis spectrum of Q-PDA-Au-DASA.

Based on a recent report[2] on the construction of DASAs by exploiting the N-content of PDA, we tested the reaction of Q-PDA surfaces with furan adduct **8**. No obvious changes with time or temperature were detected in the UV-vis spectra of the treated Q-PDA slides (Figure S5). It has been reported elsewhere[3] that the reaction of Meldrum's furan adduct with aminated surfaces shows selectivity towards secondary amines, while PDA (apart from indol-type Ns) contain mostly primary amines. This, along with the possible formation of non-absorbing closed form of the surface-bound DASA, could be responsible for the lack of pronounced absorption in the visible-region of the spectra. By XPS analysis (see Figure S13, Section S7) it is possible to show the CF_3-moiety of the molecule suggesting the occurrence of the interfacial reactions between Q-PDA and furan adduct **8**.

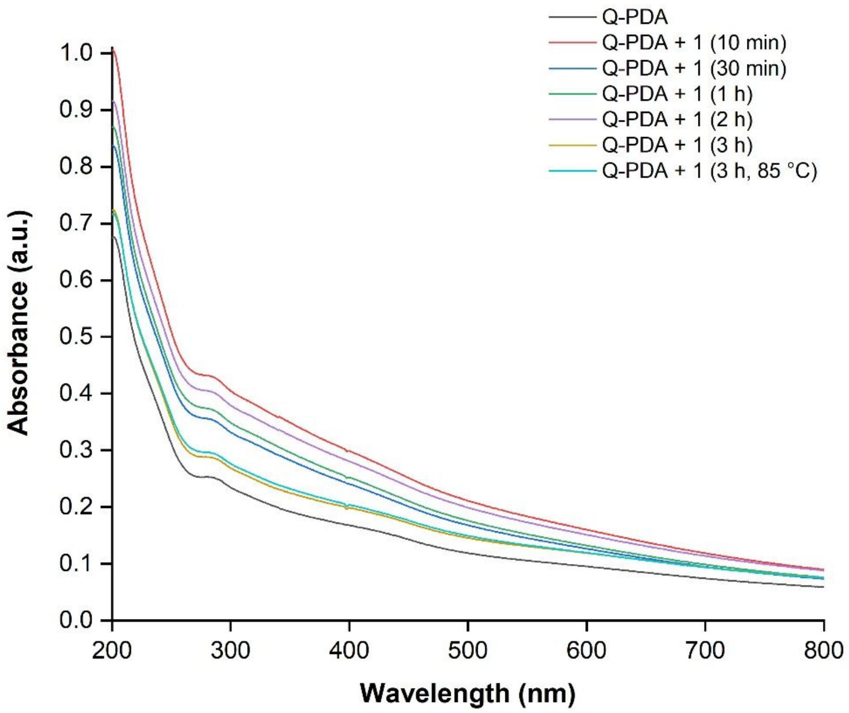

Figure S5. Solid state UV-Vis spectra monitoring the reaction of Q-PDA with furan adduct **8** (Q-PDA-DASA) after different reaction times and temperatures.

S5 NMR-experiments

S5.1 Irradiation of DASA-1

Due to the limited solubility of DASA-**1** in toluene and $CHCl_3$, NMR-scale irradiation experiments were performed in DMSO (Figure S6). Irradiation of a DASA-**1** sample in DMSO-d6 solution led to the disappearance of the signals of the O-H and vinyl-H protons from its ^{1}H-NMR spectrum. These changes are characteristic of the photochemical formation of the closed isomer of DASAs. Comparison of the resulting ^{1}H-NMR spectrum with previously reported spectra[1,4,5] of structurally similar ring-closed DASAs, it is suggested that the formation of DASA-**1**cp took place. It has to be noted, however, that the slow cyclization due to the relatively high concentration[1] necessitated longer irradiation times (in total 48 h), which could lead to decomposition to some extent.

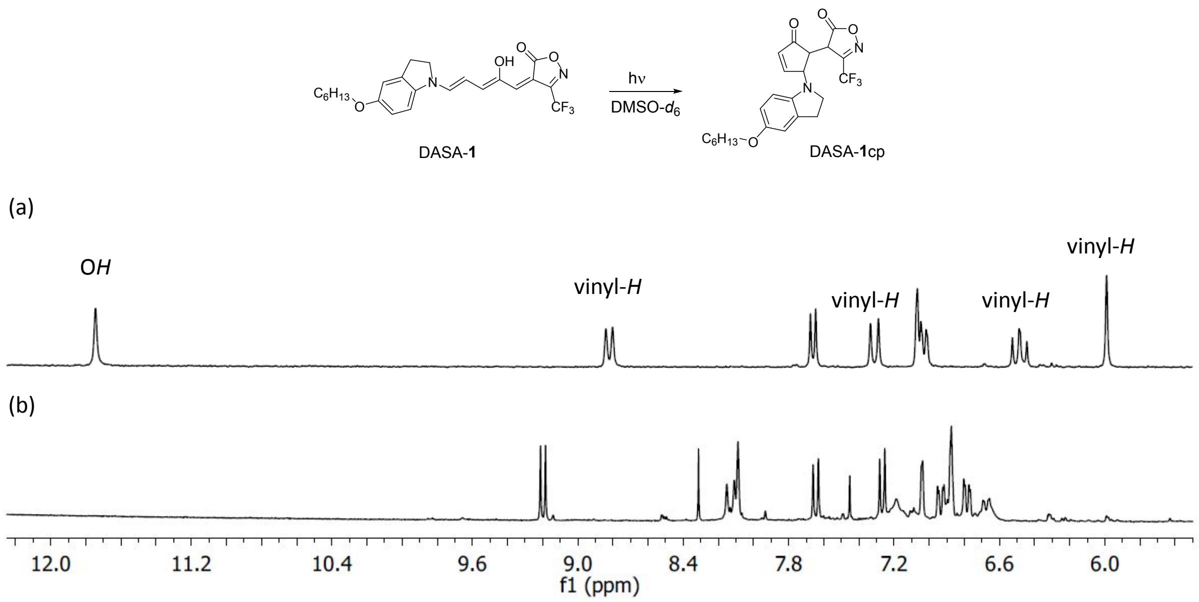

Figure S6. (a) Partial ^{1}H NMR spectrum of DASA-**1** in DMSO-d6; (b) partial ^{1}H NMR spectrum of the same sample after a total 48 h irradiation with a 620-630 nm (red) LED.

S5.2 Stability of DASA-1-SH

As a control, we synthesized DASA-**1**-SH from alkylthiolated indoline **5** and acceptor **8** (Figure S7). The synthesis conditions were identical to the conditions of making DASA-**1**. DASA-**1**-SH has limited solubility in toluene and $CHCl_3$, like DASA-**1**. To check the stability/reactivity of this compound, we submitted it to conditions that are generally used for gold surface functionalization with thiolated molecules (40°C, 12h) and monitored the changes with ^{1}H NMR spectroscopy (Figure S8). Under these conditions, the precipitation of a dark material was observed, which led to the disappearance of the molecule from solution.
We also tested the behavior of DASA-**1**-SH in MeOH solution. It has been described that in MeOH the cyclization/thiol Michael addition sequence is favored.[6] Indeed, the ^{1}H NMR spectrum of DASA-**1**-SH in CD_3OD no OH-proton signal was observed, suggesting its spontaneous conversion to the cyclopentadienone form (Figure S9). Furthermore, the signal-rich spectrum suggests that further transformation (e.g. thiol attack on the cyclopentadienone moiety) could take place.

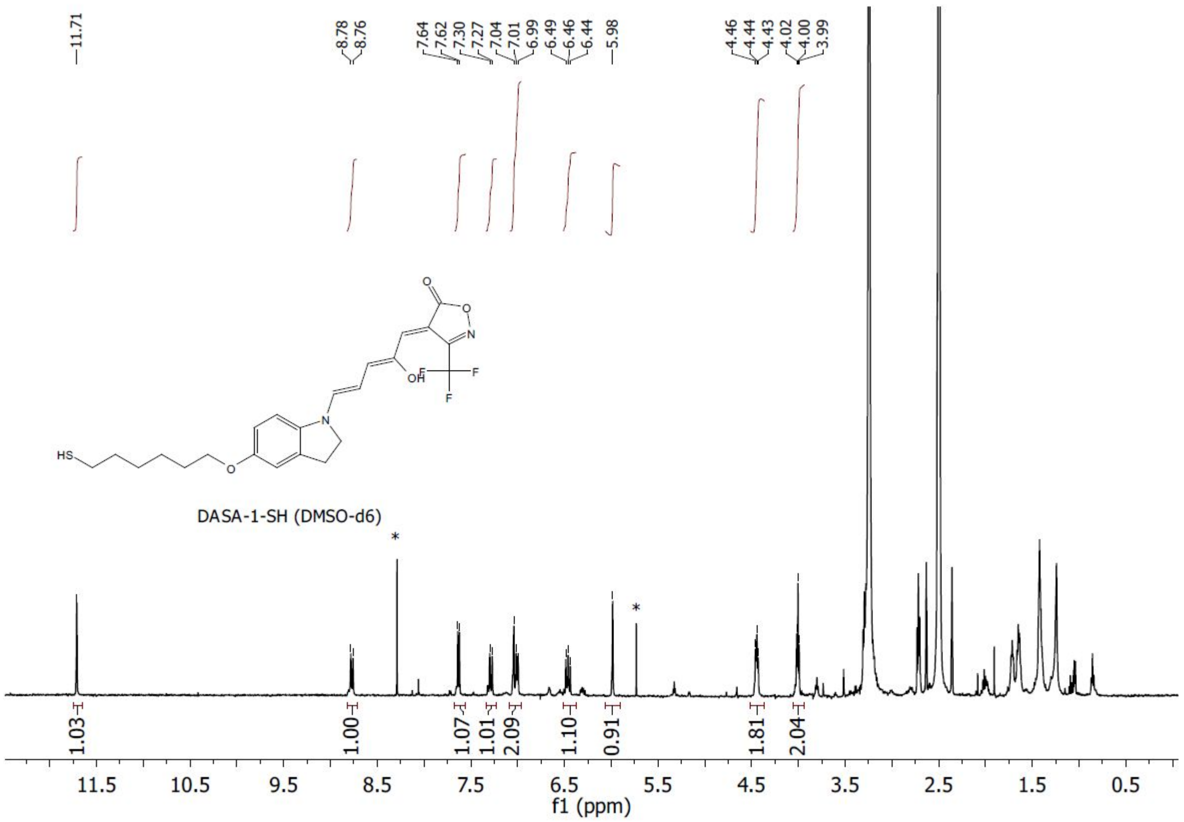

Figure S7. ^{1}H NMR spectrum of DASA-**1**-SH in DMSO-d6 (500 MHz).

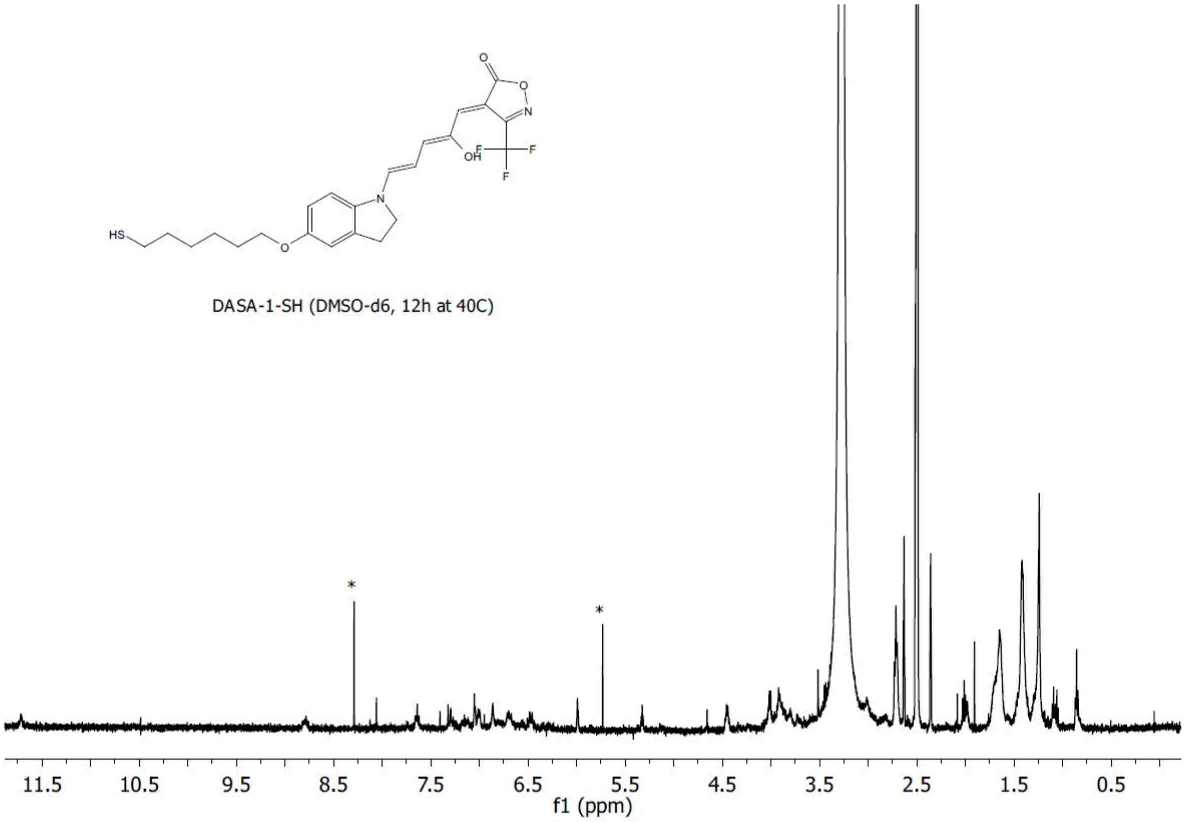

Figure S8. ^{1}H NMR spectrum of DASA-**1**-SH in DMSO-d6 (500 MHz) after 12h at 40°C.

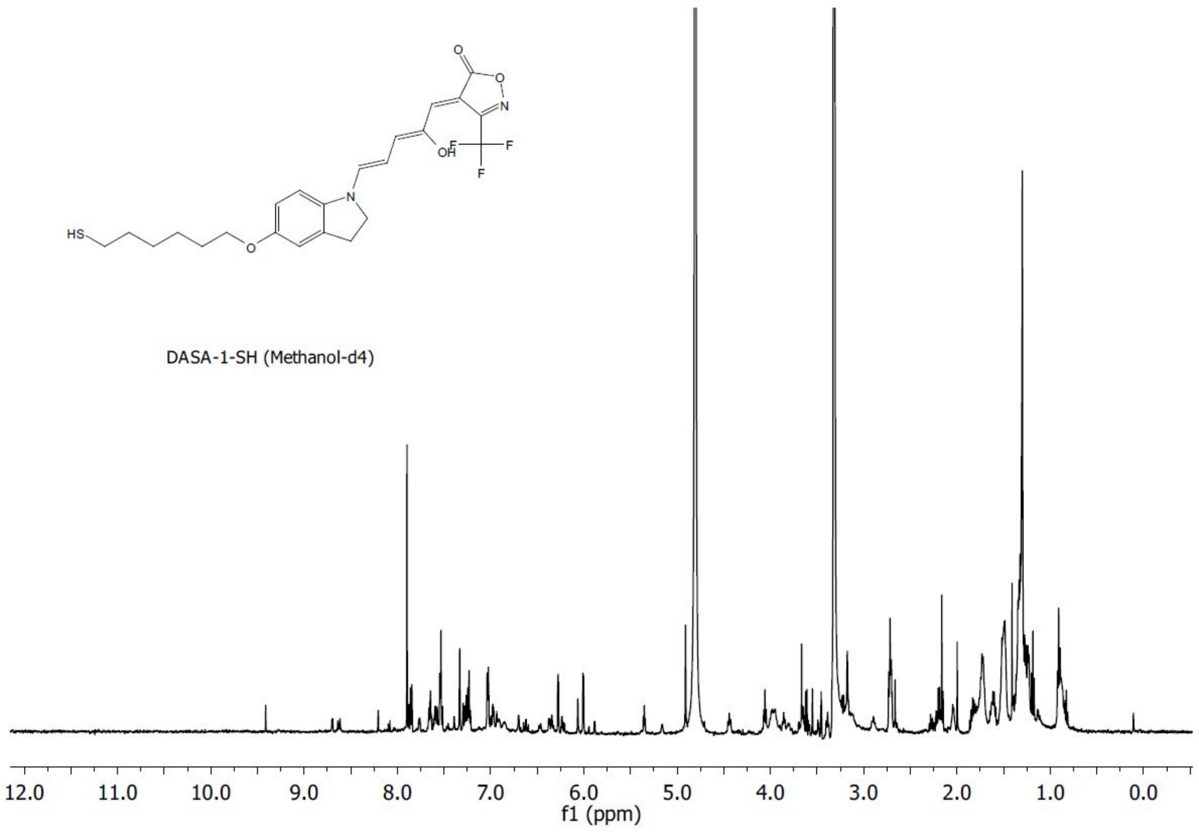

Figure S9. ^{1}H NMR spectrum of DASA-**1**-SH in CD_3OD (500 MHz).

Based on these experiments, in line with the results of previous reports,[6,7] we conclude that DASA-**1**-SH is possible to prepare, however it is unlikely to be used successfully in direct surface modifications due to its lack of solubility and, importantly, stability under assembly conditions.

S6 Contact angle measurements

The contact angle of a water droplet was measured to characterize the different wettability of the different sample surfaces. Contact angles (maximum advancing (θ_A) and minimum receding (θ_R) angles) were determined by increasing and decreasing the volume of the drop using a motor-driven Hamilton syringe. Contact angle of a sessile droplet that was deposited by the Hamilton syringe on the sample surface was determined by capturing the image from the side view with a high-resolution camera.

During wetting measurements, a 5 μL water droplet was placed on the sample surface by a Hamilton syringe. Experience has shown that the drop started to spread to a greater or lesser extent when placed on the surface. Therefore, in these cases, the change of the drop contour

was followed for 5 min without modifying the volume (Figure S10). The contact angle changing was monitored by the camera and the contact angle was fitted image by image.

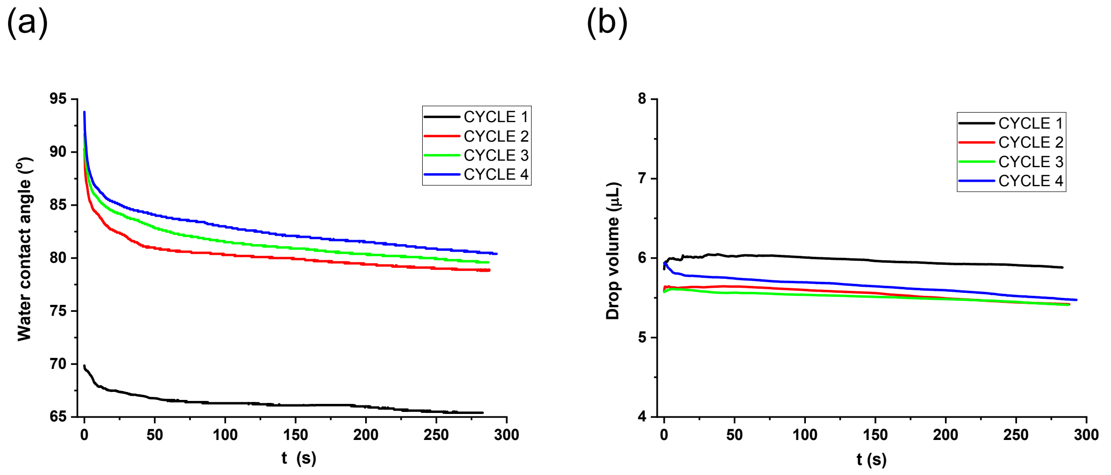

Figure S10. (a) Time-dependence of water contact angle on Q-PDA-Au-DASA surfaces in the consecutive washing/drying/measurement cycles. Note that in the first cycle (black line) no drying step was involved, the as-prepared slide was measured. (b) The time dependence of the drop-volume during the contact angle measurement.

Wetting tension was detected on the sample plate upon immersing it into water and recording the force acting along the three-phase contact line using a Sigma Force Tensiometer 700. Keeping the plate in a fixed position continuous increase of force and hence decrease of contact angle was observed indicating water spreading and approaching a steady value after approximately 5 min.

S7 XPS analyses

Survey spectra were recorded in the kinetic energy range 150 – 1300 eV with 0.5 eV steps. Photoelectron lines of the main constituent elements, i.e., the O1s, N1s, C1s, F1s, S2p and Au4f were recorded by 0.1 eV steps. Spectra were referenced to the energy of the C1s line of the hydrocarbon type carbon, set at 284.7 ± 0.1 eV binding energy (B.E.). A Gaussian-Lorenzian peak shape (70/30 ratio) was used for peak decomposition. Quantitative analysis, based on peak area intensity after removal of the Shirley-type background, was performed by the XPS MultiQuant program[8], using experimentally determined photo-ionization cross-section data of Evans et al.[9] and asymmetry parameters of Reilman et al.[10] In all cases the

conventional infinitely thick layer model was employed, where all components are supposed to be homogeneously distributed within the sampling depth detected by XPS.

Photoelectron spectra of the samples are complex, due to the several types of chemical states found on the surface.

The high-resolution spectra of the Q-PDA-Au-DASA sample are shown in Figure S11. The C1s, O1s and N1s lines are complex, indicating presence of several chemical states of these elements. Due to the complexity of the Q-PDA-Au base of the molecules[11], containing the same elements (C, O, N), complete resolution of the states cannot be expected. E.g., although the O1s line was decomposed, its components could not be reliably assigned to the various chemical states.

The total composition of the samples are shown in Table S1. It demonstrates that all expected hetaroatoms (S, F) are present on the surface in well detectable quantity. The concentrations of other elements (C, O, N, Au) are highly depending on the actual coverage of the Au upon the consecutive modification steps. The composition of the various chemical states (Table S2) give more information on the bound molecules. The ratio of the F, C4 and S species are very close to the expected 3:1:1 values, proving that the whole molecule is present on the surface. Although the other components cannot be distinguished from the constituents of the Q-PDA base, the oxygen balance (the ratio of the measured and calculated, i.e., required by the other elements at the supposed stoichiometry, oxygen concentration) is close to one, which also confirms the assignation of the states (Table S3).

Table S1. Total surface composition (atomic %) of the modified surfaces.

Sample	O	F	N	C	S	Au
Q-PDA-Au-DASA	17.3	6.3	4.7	68.0	1.8	1.9
Q-PDA-Au-Ind	20.5		5.7	71.1	0.7	2.0
Q-PDA-DASA	19.9	4.3	5.9	70.0		

Table S2. Surface composition (atomic %) of the chemical states of modified surfaces.

Sample	O	F	N1	N2	S
Q-PDA-Au-DASA	17.3	6.3	2.4	2.3	1.9
Q-PDA-Au-Ind	20.5		5.7	0.0	2.0
Q-PDA-DASA	19.9	4.3	3.8	2.1	
	**C1**	**C2**	**C3**	**C4**	**Au**
Q-PDA-Au-DASA	40.9	19.0	6.0	2.0	1.8
Q-PDA-Au-Ind	46.6	18.6	5.9		0.7
Q-PDA-DASA	42.5	19.5	6.4	1.6	

Table S3. Assignation of chemical states.

Component	**Binding energy** (eV)	Chemical states
O		
N1	399.9 ± 0.2	N–C
N2	400.8 ± 0.2	O–N–C
C1	284.7 ± 0.1	C–C, C–H
C2	286.2 ± 0.1	C–OH, O–C–O, C–X *heteroatoms*
C3	288.3 ± 0.1	O–C=O
C4	292.4 ± 0.1	C–F
S	163.5 ± 0.1	$–SH_2$
Au	88.1 ± 0.1	metallic *(charged)*
F	688.3 ± 0.1	F–C

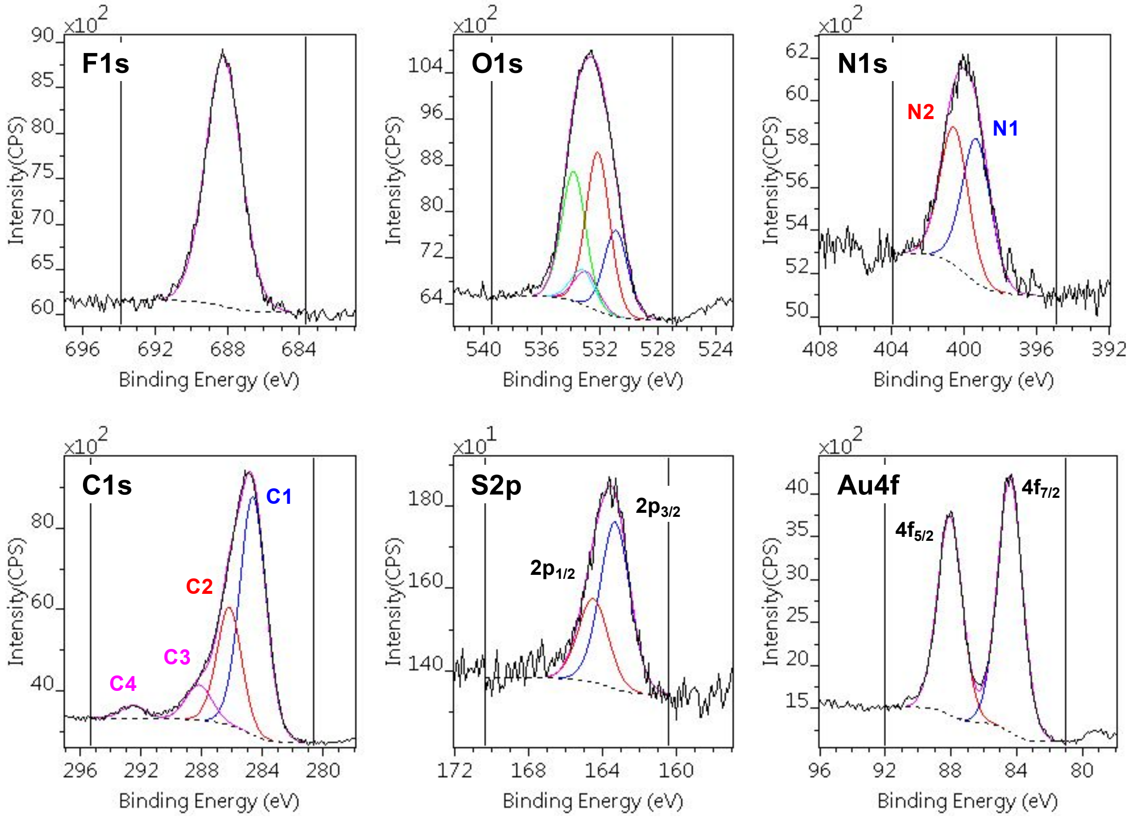

Figure S11. High resolution X-ray photoelectron spectra of the Q-PDA-Au-DASA sample.

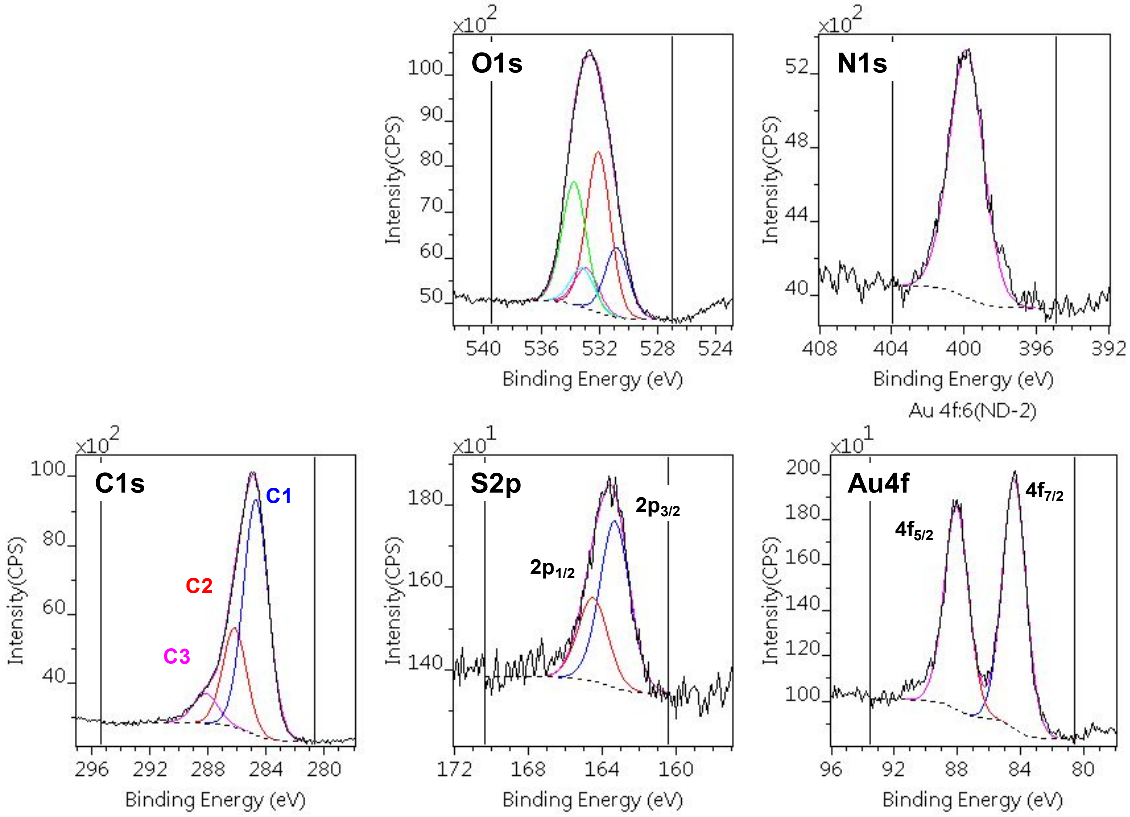

Figure S12. High resolution X-ray photoelectron spectra of the Q-PDA-Au-Ind sample.

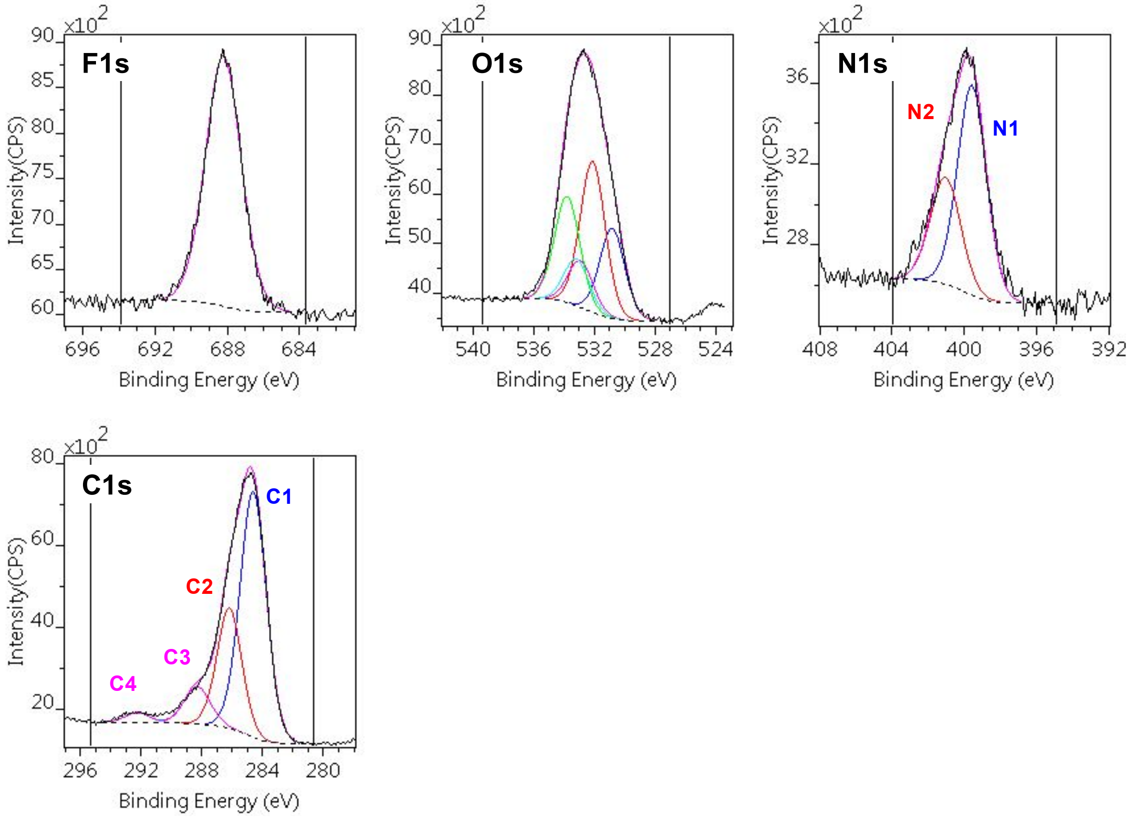

Figure S13. High resolution X-ray photoelectron spectra of the Q-PDA-DASA sample.

S8 Computational details

All calculations were performed with the Gaussian 09[12] package using the B3LYP[13] hybrid functional.

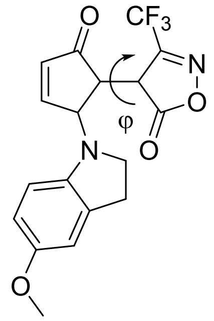

Figure S14. The structure of DASA-**1**cp' having only a methoxy group on the aryl ring. Potential energy surface scans were preformed around bond φ.

At first, a potential energy surface scans2 around the C-C bond connecting the isoxazolone and cyclopentenone rings (φ, Figure S14) were performed in gas-phase and with implicit water model using 6-31G* [14] basis set. These scans were performed with +10° and -10° steps and at each step with a maximum of 20 cycles which gave similar energy profiles (Figure S15). The energy surface scans provided two conformers at very similar φ degrees (30° and 180°). The rotamer where the CF_3 group of the isoxazolone ring is oriented towards the C=O group of the cyclopentenone is denoted as DASA-**1**cp'(CO,CF_3) (φ~180°), while the one where the two C=O groups are in proximity is denoted as DASA-**1**cp'(CO,CO) (φ~30°). These conformers were further optimized with the 6-311+G(d,p)[15] basis set. Analytical hessians were computed to confirm that the structures are minima. Both in gas-phase and in solvent model the DASA-**1**cp'(CO,CO) rotamer were the more stable by 0.2 kcal/mol and by 1 kcal/mol respectively.

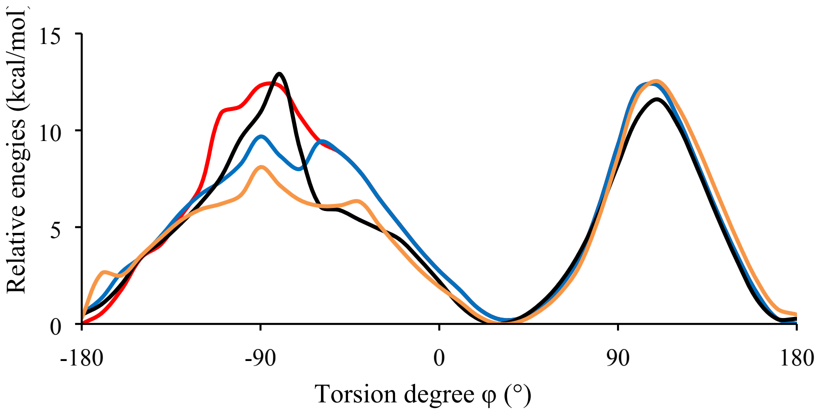

Figure S15. The potential energy surface scans of DASA-**1**cp' around the C-C bond: in gas-phase with +10° (red) and -10° (blue) steps and in solvent model with +10 degree (green) and -10 degree (black) steps.

Cartesian coordinates and absolute electronic energies

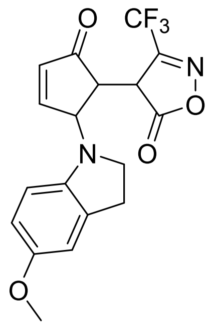

DASA-**1**cp'(CO,CF_3)

In gas-phase

E_{el}= -1405.17522594 Hartree

-881760.955243909 kcal/mol

	X	Y	Z
C	4.604149	0.376231	0.292073
C	3.727667	1.445894	0.491470
C	2.425511	1.415255	-0.005390
C	1.996849	0.287257	-0.708024
C	2.877707	-0.778494	-0.914320
C	4.177381	-0.748597	-0.427609
N	0.756702	0.029749	-1.337269
C	0.711119	-1.414835	-1.653827
C	2.192955	-1.821475	-1.769042
C	-0.477577	0.695513	-0.974897
C	-0.501444	2.161626	-1.368383
C	-1.069120	2.959004	-0.456214
C	-1.484990	2.165960	0.713221
C	-0.906304	0.747920	0.530714
C	-1.842801	-0.320645	1.130916
C	-2.954778	-0.904284	0.311721
N	-2.929073	-2.178082	0.220891
O	-1.809858	-2.660465	0.922844
C	-1.117320	-1.603981	1.508295
O	-2.163048	2.514735	1.654090
O	-0.134106	-1.774778	2.155235
O	5.853808	0.518420	0.835915
C	6.777596	-0.548947	0.694030
C	-4.121178	-0.161254	-0.294461
F	-4.920309	-0.955923	-1.007821
F	-3.678787	0.825170	-1.113364
F	-4.854342	0.422184	0.671761
H	4.082125	2.304056	1.049430
H	1.779387	2.268166	0.155789
H	4.837619	-1.588145	-0.603722
H	0.235412	-1.985693	-0.844598
H	0.144342	-1.585093	-2.571663
H	2.537414	-1.745919	-2.807372
H	2.364272	-2.846422	-1.435129
H	-1.262112	0.201619	-1.559470
H	-0.102664	2.470732	-2.327390
H	-1.246740	4.023079	-0.534494
H	0.007918	0.717281	1.136437
H	-2.253553	0.127010	2.048312
H	7.683129	-0.230578	1.208322
H	6.403075	-1.468453	1.158157
H	7.011029	-0.742372	-0.359447

In water solvent model

E_{el}= -1405.19341980 Hartree

-881772.372065866 kcal/mol

	X	Y	Z
C	4.607890	0.380896	0.338121
C	3.711705	1.427425	0.570193
C	2.411052	1.393458	0.062637
C	2.006648	0.284857	-0.685117
C	2.910756	-0.758222	-0.925576
C	4.206013	-0.724435	-0.427390
N	0.779131	0.024940	-1.326768
C	0.761278	-1.405774	-1.708705
C	2.251549	-1.777684	-1.827674
C	-0.468116	0.681663	-0.993321
C	-0.500928	2.142279	-1.401125
C	-1.087091	2.943731	-0.502193
C	-1.508173	2.159669	0.663996
C	-0.915361	0.747069	0.507262
C	-1.835116	-0.331185	1.114747
C	-2.973405	-0.907799	0.327173
N	-2.965308	-2.181340	0.245564
O	-1.825981	-2.668221	0.920446
C	-1.108540	-1.617096	1.468378
O	-2.204422	2.514095	1.597052
O	-0.103234	-1.799238	2.084893
O	5.855392	0.520184	0.897031
C	6.807812	-0.521691	0.693261
C	-4.144670	-0.157098	-0.260513
F	-4.971887	-0.954088	-0.942590
F	-3.714804	0.812796	-1.098833
F	-4.851730	0.444620	0.716725
H	4.041715	2.274489	1.160295
H	1.7518	2.230566	0.251108
H	4.882844	-1.543684	-0.632815
H	0.287603	-2.017807	-0.929161
H	0.205338	-1.544555	-2.636881
H	2.602294	-1.650294	-2.858373
H	2.443596	-2.811750	-1.538018
H	-1.237379	0.173005	-1.583988
H	-0.096894	2.449499	-2.358090
H	-1.270182	4.005425	-0.596252

H	-0.008491	0.740381	1.123599
H	-2.225906	0.096490	2.049845
H	7.708332	-0.209852	1.219302
H	6.455022	-1.470646	1.109375
H	7.035479	-0.651365	-0.369545

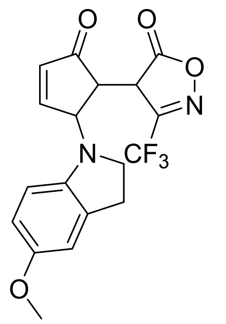

DASA-**1**cp'(CO,CO)

In gas-phase

E_{el}= -1405.17554927 Hartree

-881761.15813659 kcal/mol

	X	Y	Z
C	-4.258255	0.647975	0.455098
C	-3.343636	0.390917	1.480152
C	-2.228386	-0.417861	1.267897
C	-2.026469	-0.971436	0.001396
C	-2.946366	-0.719083	-1.021398
C	-4.062915	0.079153	-0.811311
N	-1.012391	-1.845904	-0.451507
C	-1.073350	-1.858774	-1.928212
C	-2.539054	-1.507700	-2.246090
C	0.289414	-1.952128	0.177381
C	0.260401	-2.611360	1.541931
C	1.106935	-2.059823	2.420416
C	1.802826	-0.920774	1.795243
C	1.096299	-0.639451	0.458073
C	2.059081	-0.162484	-0.644736
C	2.822265	1.112994	-0.439373
N	4.076640	1.019199	-0.644914
O	4.380221	-0.329173	-0.940627
C	3.235629	-1.096965	-0.917335
O	2.773714	-0.321269	2.203462
O	3.250722	-2.276058	-1.103190
O	-5.308946	1.463538	0.782832
C	-6.272913	1.759215	-0.215468
C	2.212773	2.463371	-0.160152
F	1.310333	2.754393	-1.133068
F	3.113705	3.442638	-0.123741
F	1.539628	2.461085	1.007566
H	-3.519240	0.839801	2.450233
H	-1.550702	-0.609692	2.089630
H	-4.761250	0.252563	-1.620023
H	-0.410369	-1.091199	-2.357849
H	-0.763616	-2.830742	-2.316416
H	-3.145584	-2.417649	-2.328568
H	-2.639123	-0.955853	-3.182634
H	0.890591	-2.599001	-0.471268
H	-0.374014	-3.471900	1.719406
H	1.319374	-2.395056	3.426703
H	0.368070	0.156252	0.646681
H	1.494457	-0.070688	-1.580768
H	-7.004034	2.411575	0.259491
H	-5.822845	2.282265	-1.067087
H	-6.775810	0.852196 -	0.570279

In water solvent model

E_{el}= -1405.19493601 Hartree

-881773.323502209 kcal/mol

	X	Y	Z
C	-4.23693400	0.61499200	0.48362900
C	-3.31610500	0.33716500	1.49747200
C	-2.19856400	-0.46589500	1.26013100
C	-2.00376700	-0.99128200 -	0.01963000
C	-2.93298900	-0.71829700	-1.03175600
C	-4.04865500	0.07398900	-0.79720700
N	-0.99195300	-1.84925400	-0.49941400
C	-1.06659700	-1.84047100	-1.98007100
C	-2.53241300	-1.47429500	-2.27856400
C	0.31138800	-1.97561200	0.12008900
C	0.28939700	-2.67277000	1.46460600
C	1.13562800	-2.14123900	2.35825500
C	1.82212000	-0.98888200	1.76396000
C	1.11869600	-0.66993900	0.43873700
C	2.07648500	-0.15076600	-0.65219500
C	2.77285600	1.15965000	-0.43163800
N	4.03583400	1.12664200	-0.60189800
O	4.40961300	-0.20567900	-0.89331500
C	3.29831300	-1.02609500	-0.89927900
O	2.79836500	-0.39434100	2.18774300
O	3.38939600	-2.20354200	-1.08083700
O	-5.29141500	1.42393800	0.83176500
C	-6.26824600	1.72773900	-0.16233100
C	2.10974400	2.48486700	-0.14385700
F	1.20724500	2.77108400	-1.10680600
F	2.98683600	3.49230200	-0.08414500
F	1.44249300	2.44464000	1.02836300
H	-3.48112800	0.76080900	2.48131000
H	-1.51649600	-0.67232100	2.07465700
H	-4.75155200	0.26363300	-1.59799700
H	-0.40039000	-1.07512300	-2.40176900
H	-0.77146700	-2.81066100	-2.38165300
H	-3.14523500	-2.37709500	-2.38453300
H	-2.63522200	-0.89494800	-3.19728200
H	0.90718800	-2.60390000	-0.55051200
H	-0.33959200	-3.54036100	1.62385600
H	1.34485100	-2.50523400	3.35501200
H	0.38509200	0.11390300	0.65111600

H	1.52620000	-0.08572100	-1.59847700
H	-7.00198000	2.36651900	0.32599900
H	-5.82458100	2.26542700	-1.00623900
H	-6.76182200	0.82096300	-0.52597700

S9 NMR spectra

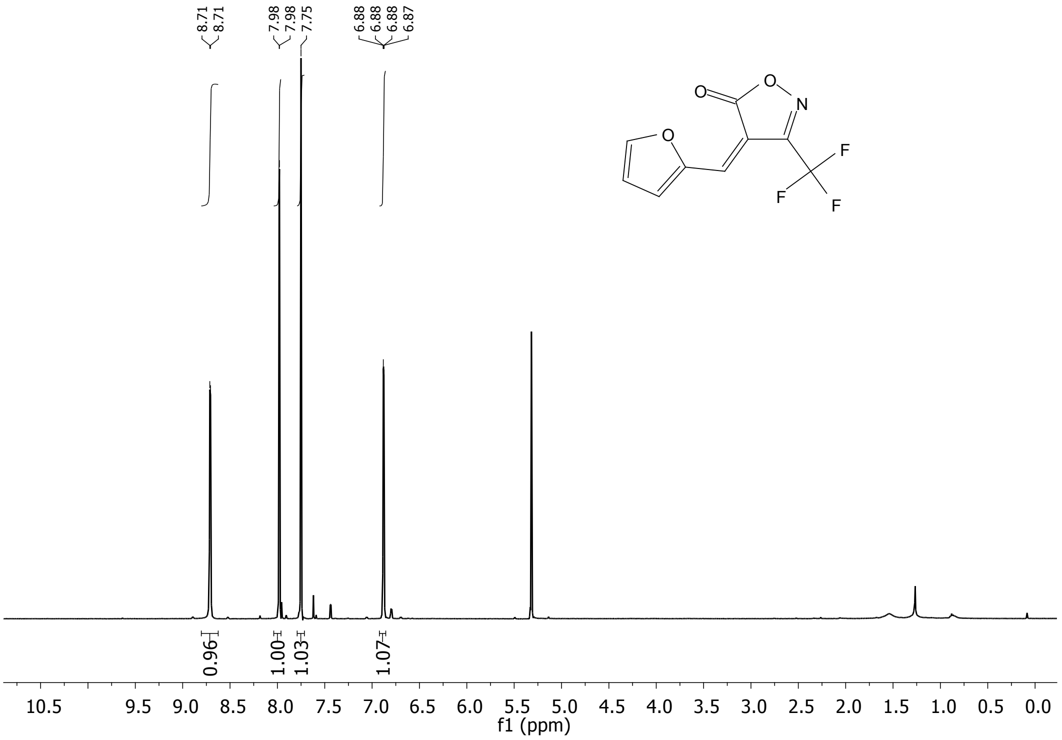

Figure S16. ^{1}H NMR spectrum of **8** in CD_2Cl_2 (500 MHz).

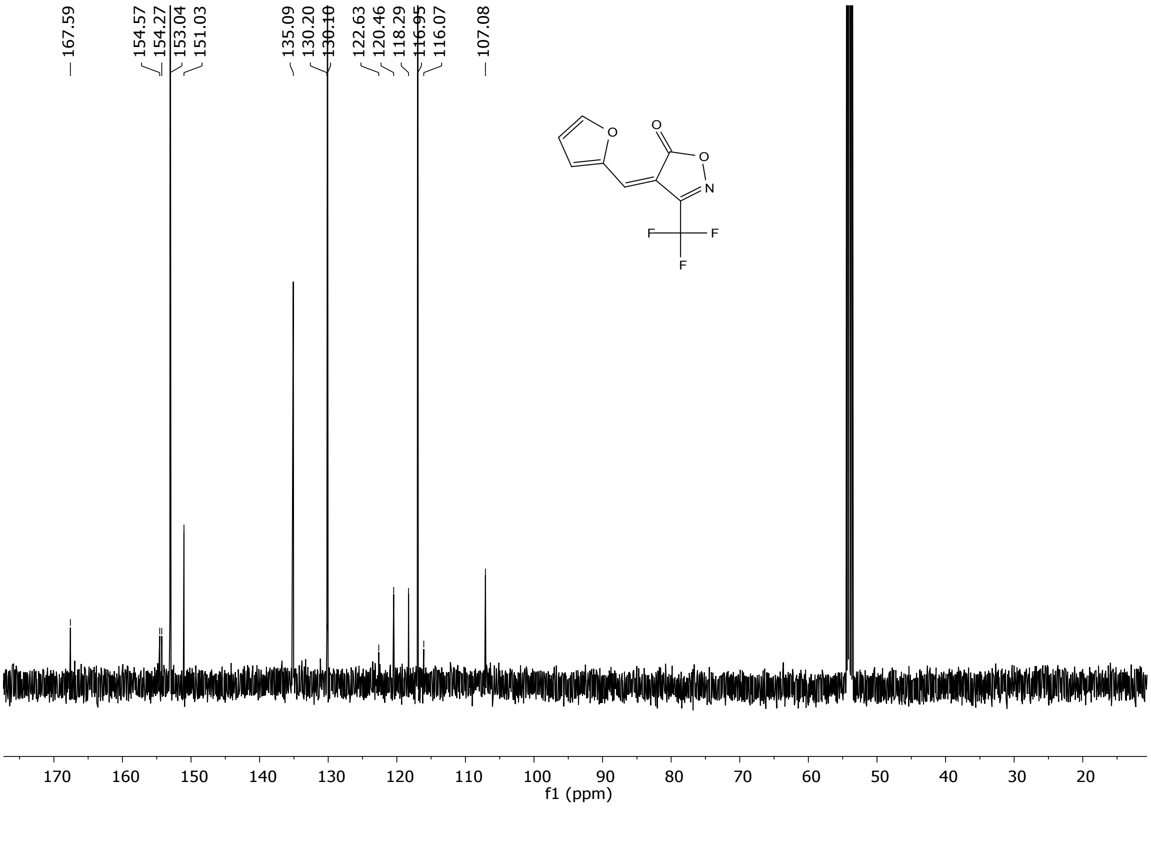

Figure S17. ^{13}C NMR spectrum of **8** in CD_2Cl_2 (500 MHz).

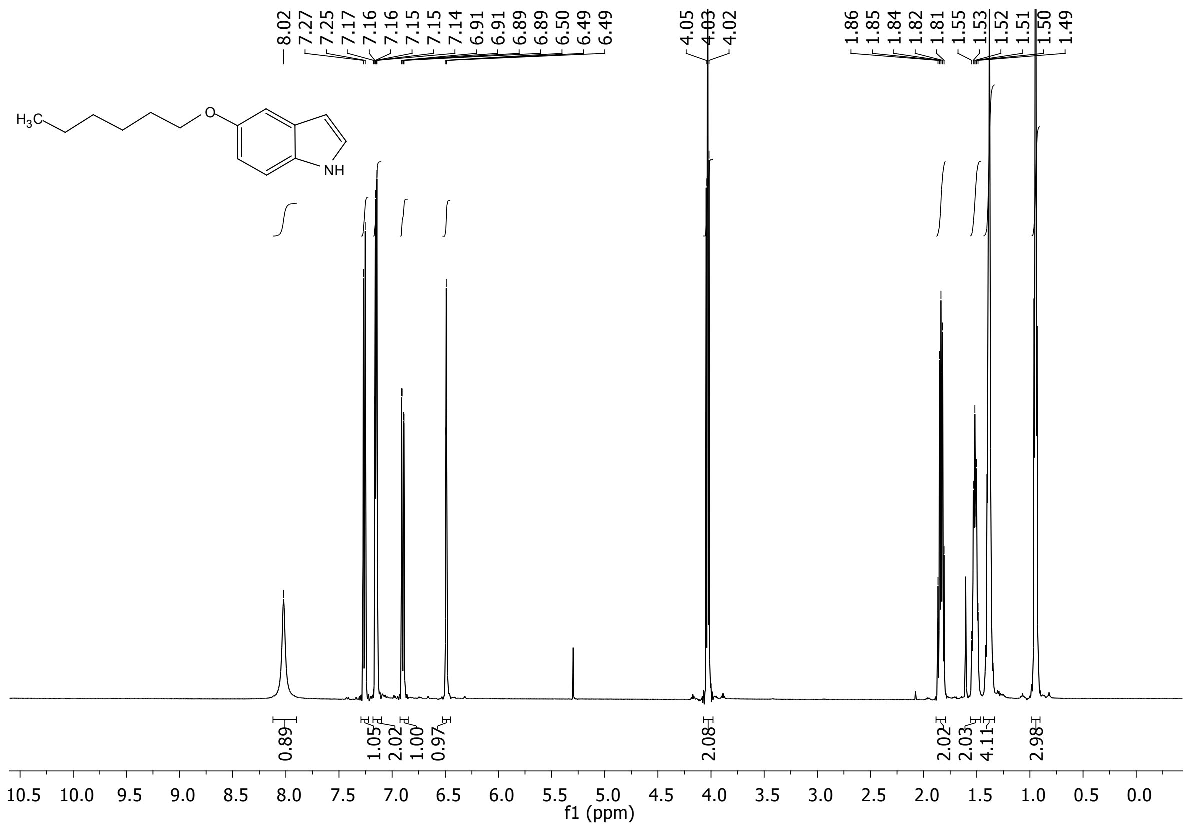

Figure S18. ^{1}H NMR spectrum of **6** in $CDCl_3$ (500 MHz).

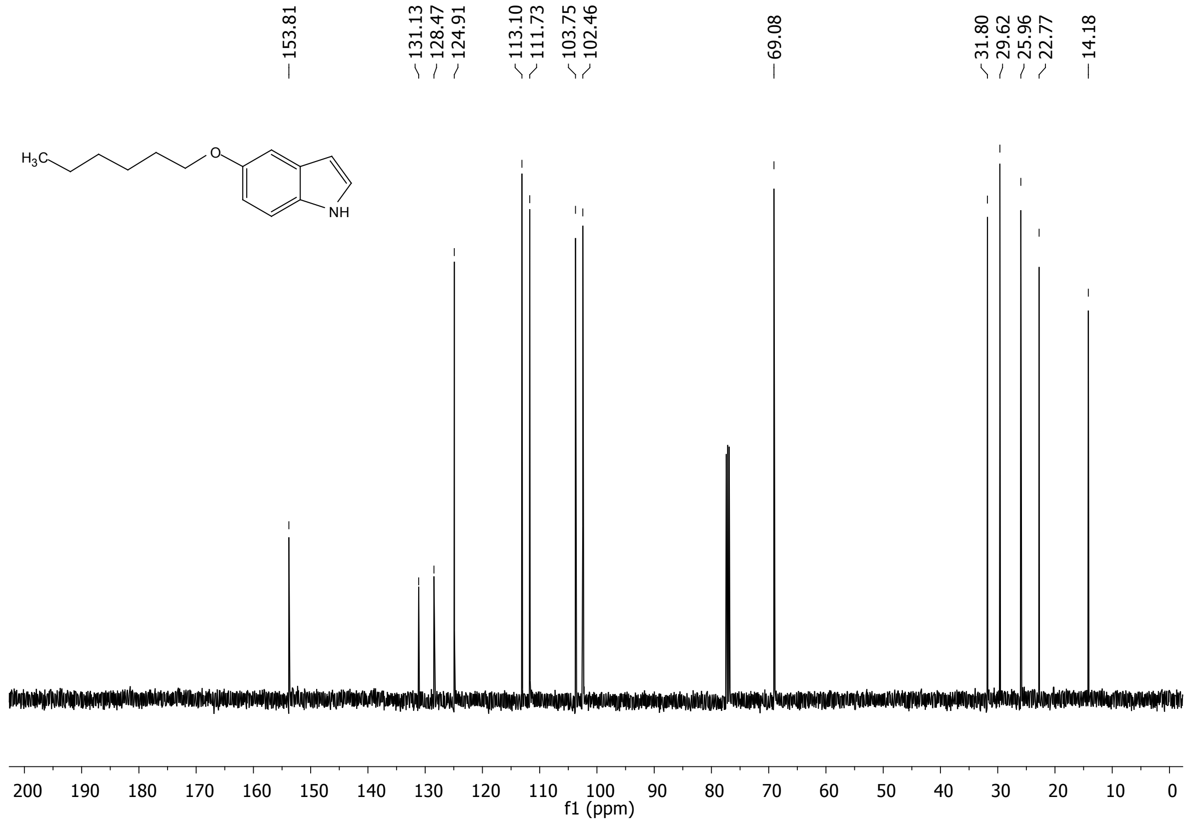

Figure S19. ^{13}C NMR spectrum of **6** in $CDCl_3$ (500 MHz).

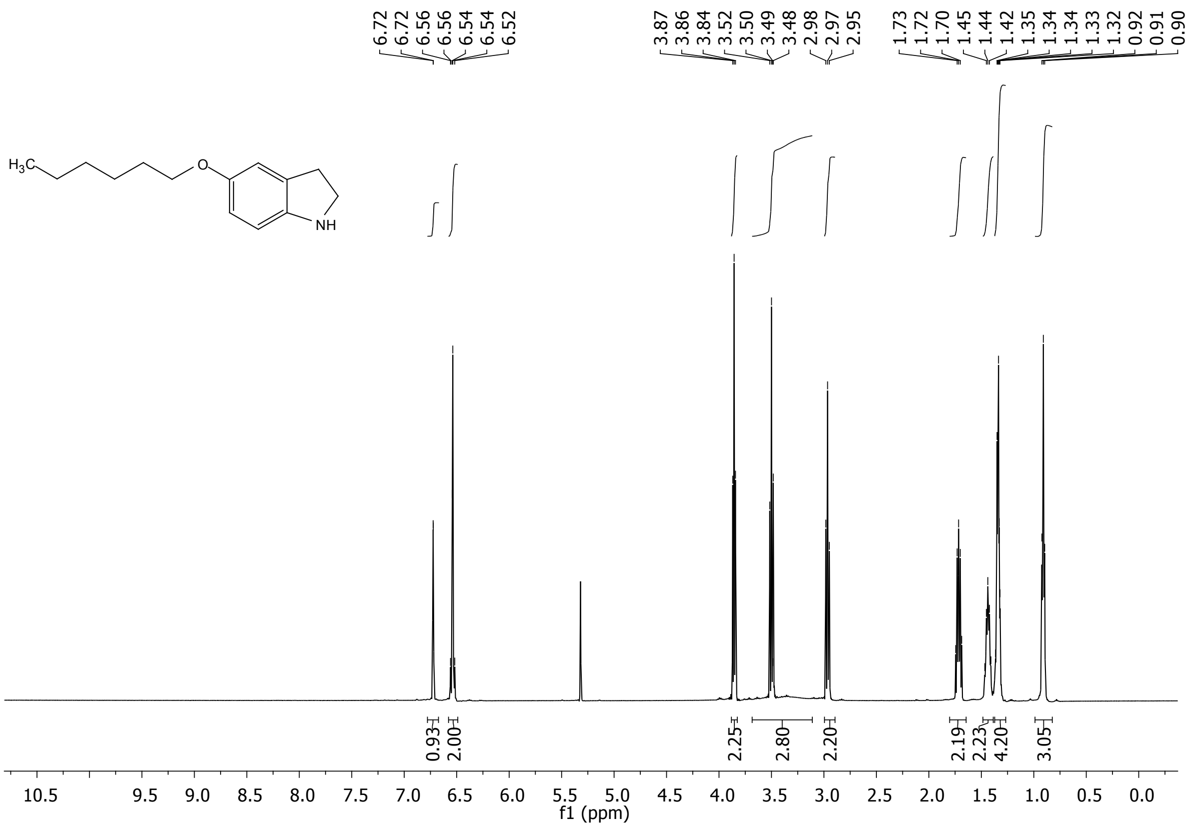

Figure S20. ^{1}H NMR spectrum of **7** in CD_2Cl_2 (500 MHz).

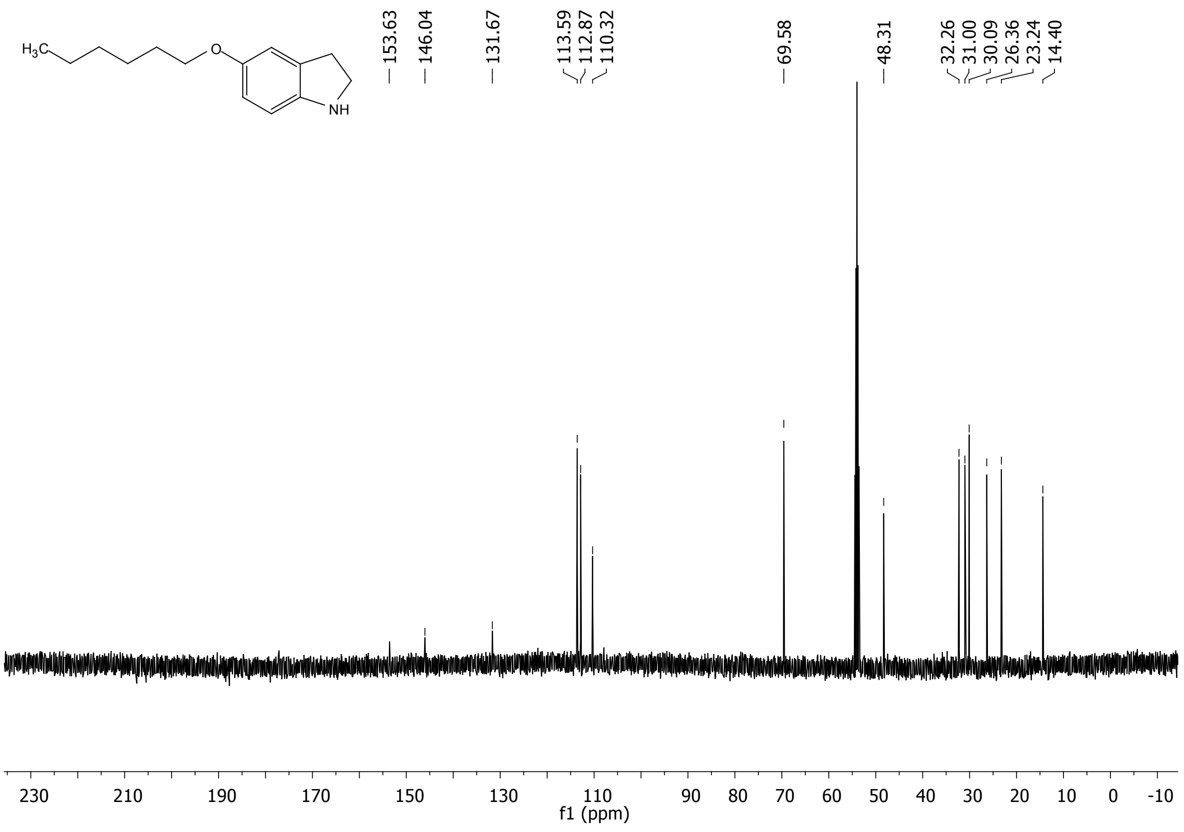

Figure S21. ^{13}C NMR spectrum of **7** in CD_2Cl_2 (500 MHz).

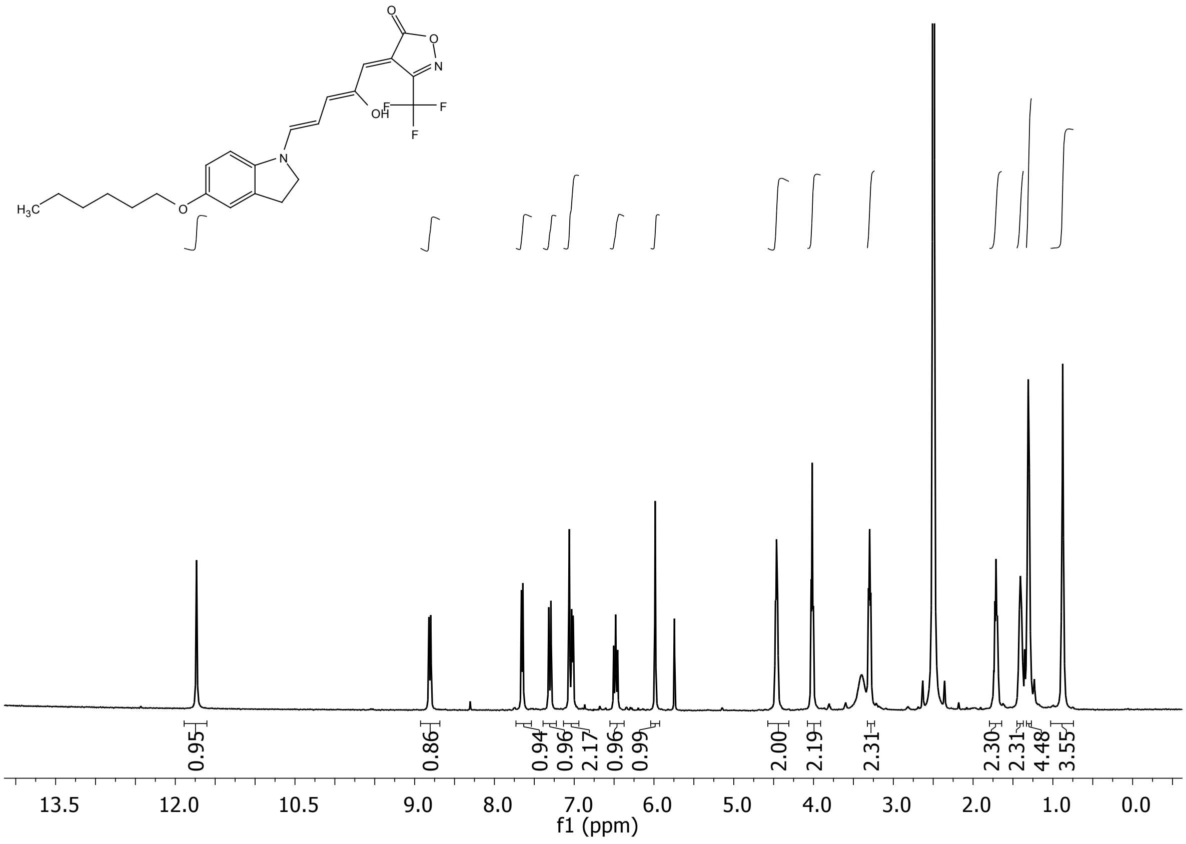

Figure S22. ^{1}H NMR spectrum of DASA-**1** in DMSO-d_6 (500 MHz).

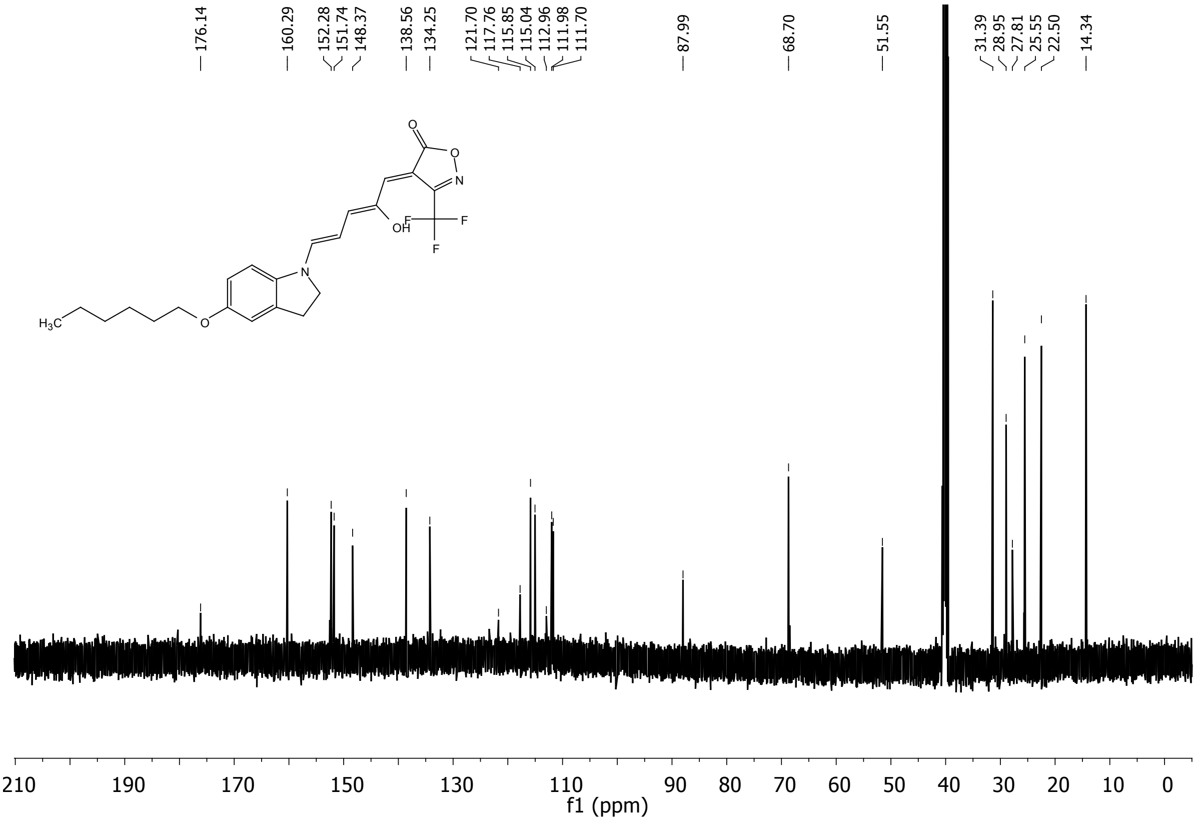

Figure S23. ^{13}C NMR spectrum of DASA-**1** in DMSO-d_6 (500 MHz).

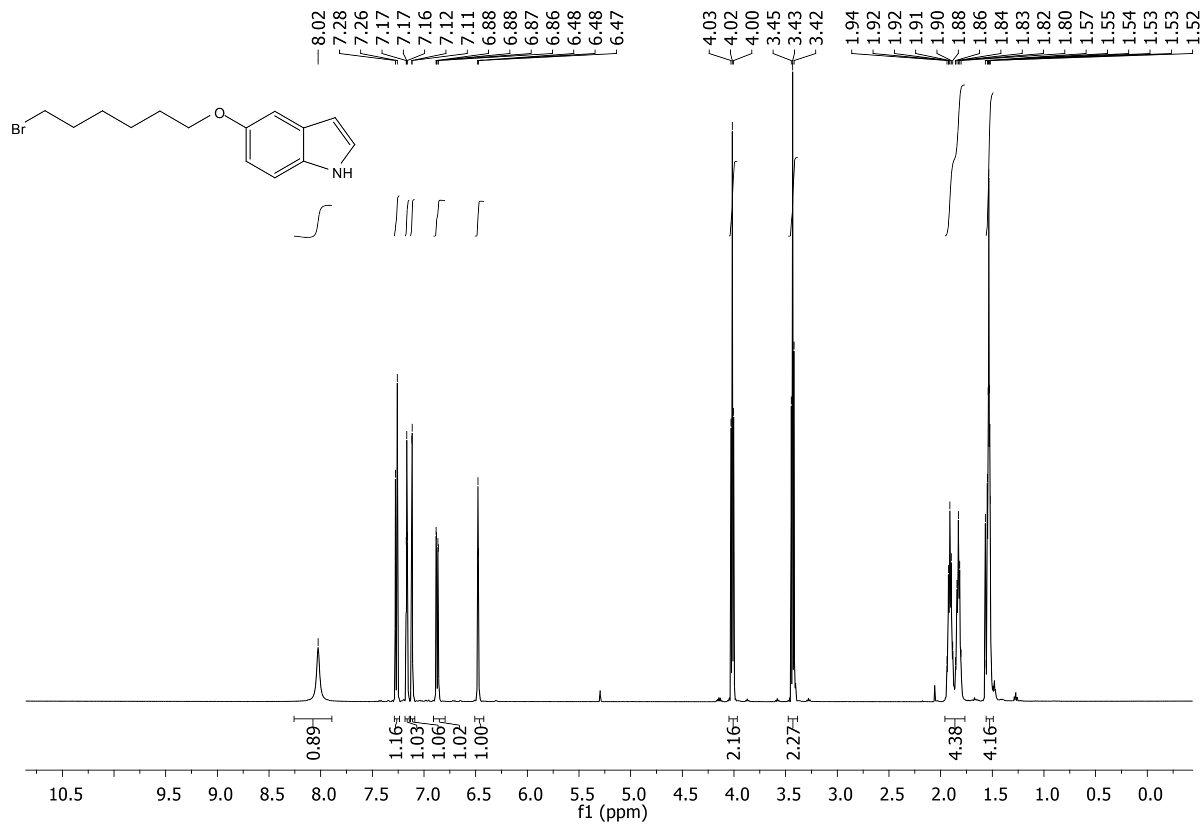

Figure S24. ^{1}H NMR spectrum of **2** in $CDCl_3$ (500 MHz).

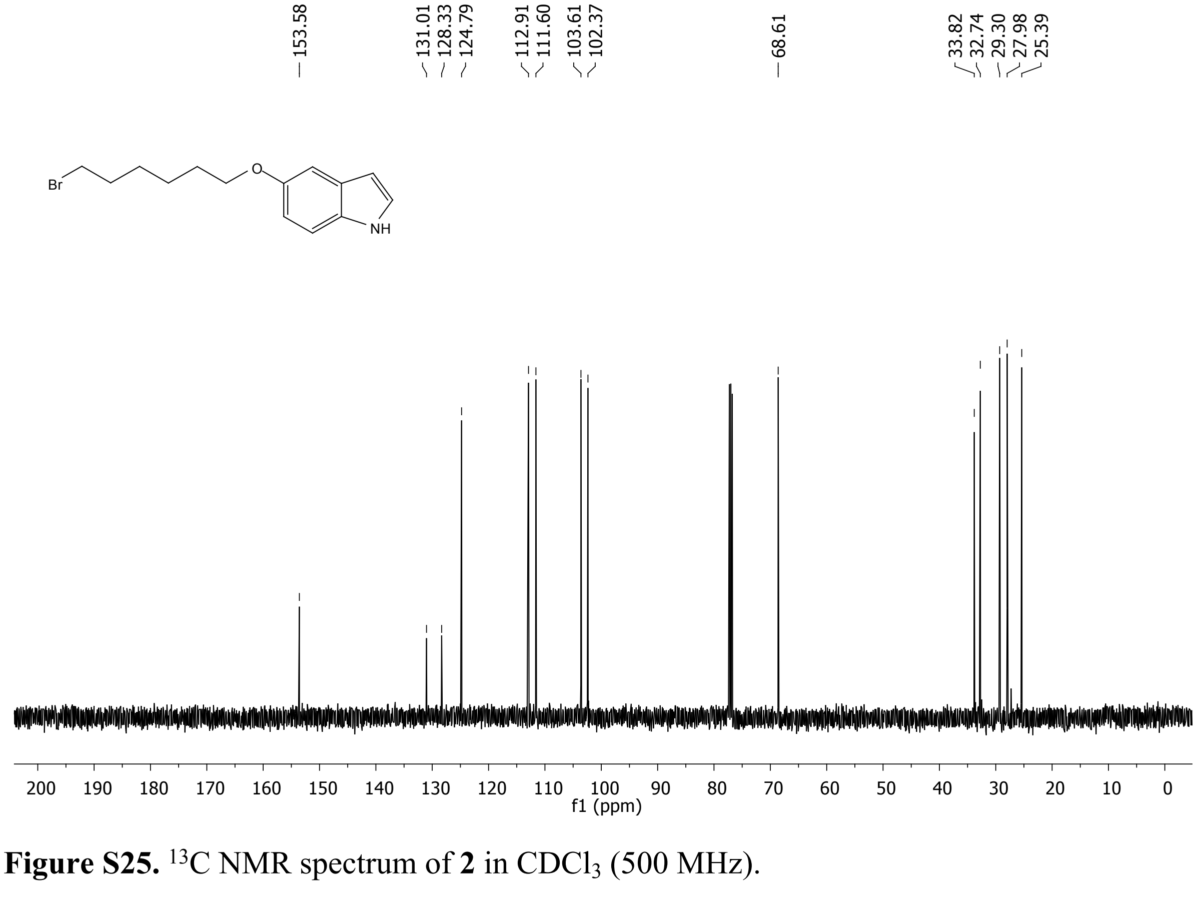

Figure S25. ^{13}C NMR spectrum of **2** in $CDCl_3$ (500 MHz).

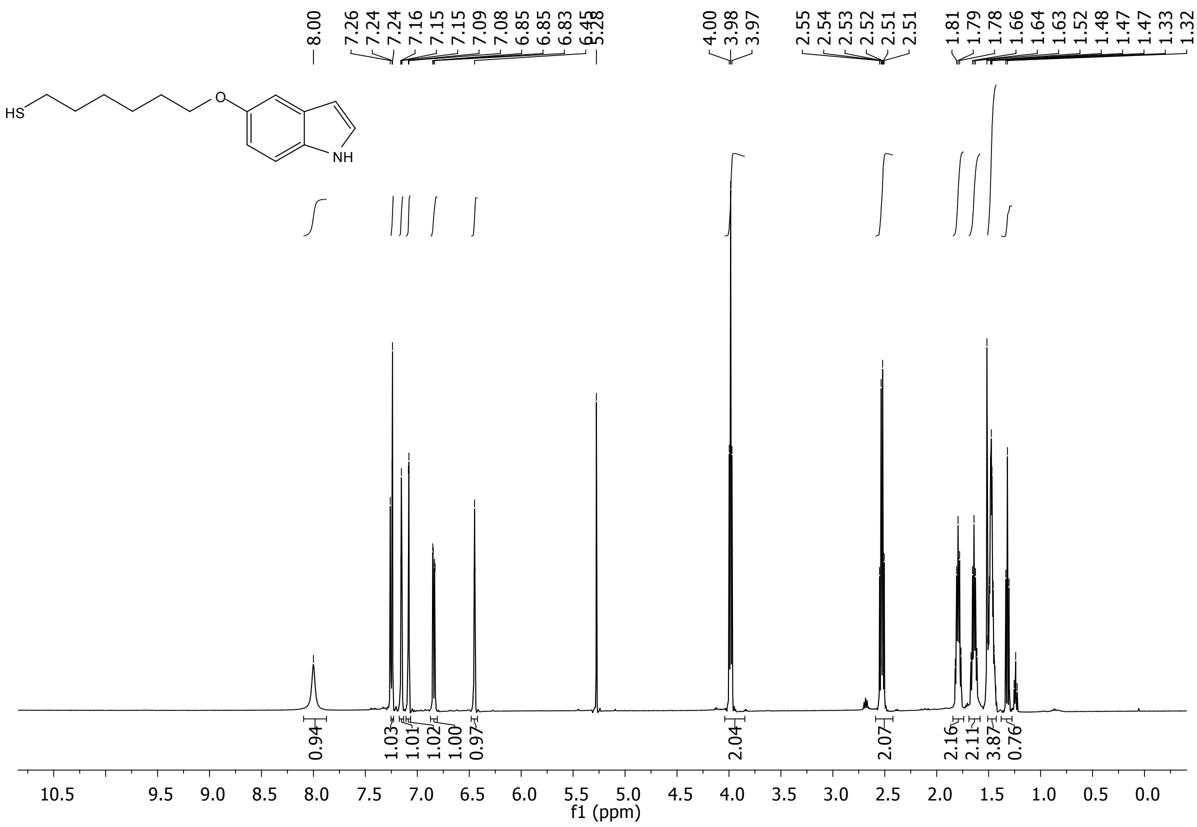

Figure S26. ^{1}H NMR spectrum of **4** in $CDCl_3$ (500 MHz).

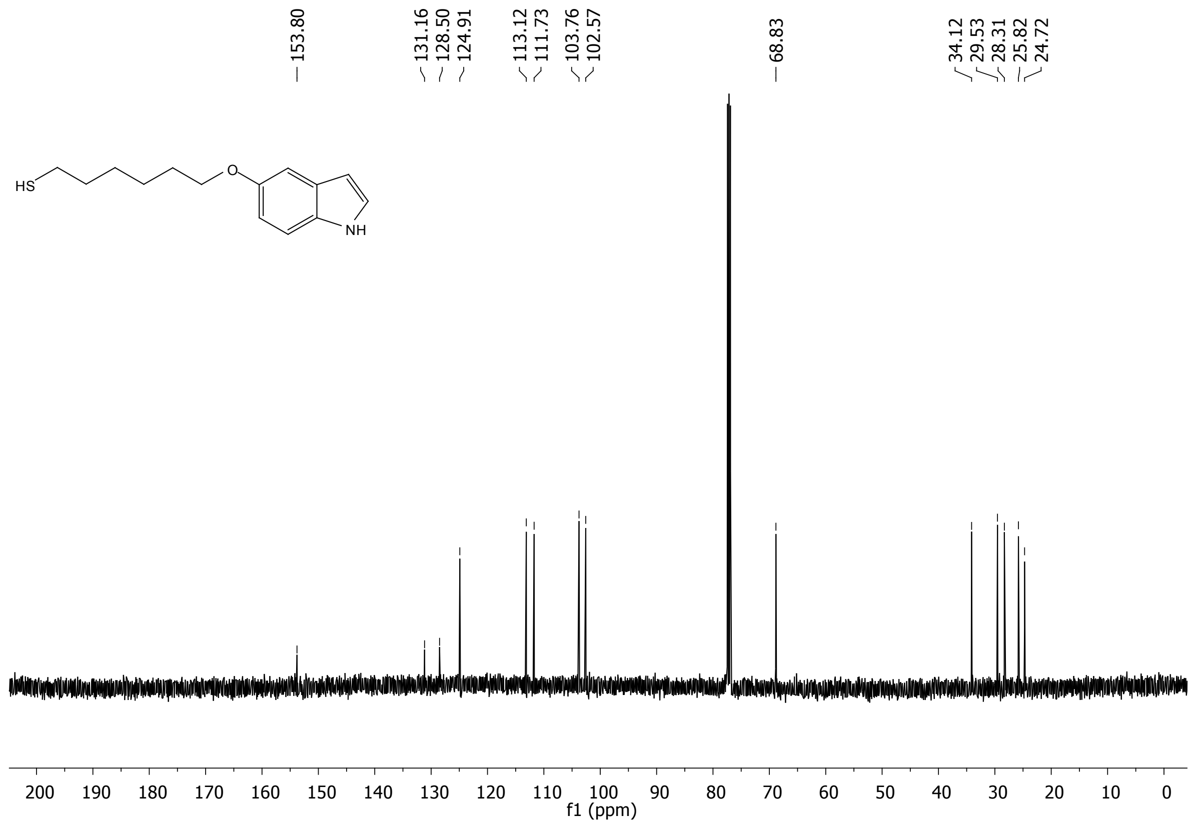

Figure S27. ^{13}C NMR spectrum of **4** in $CDCl_3$ (500 MHz).

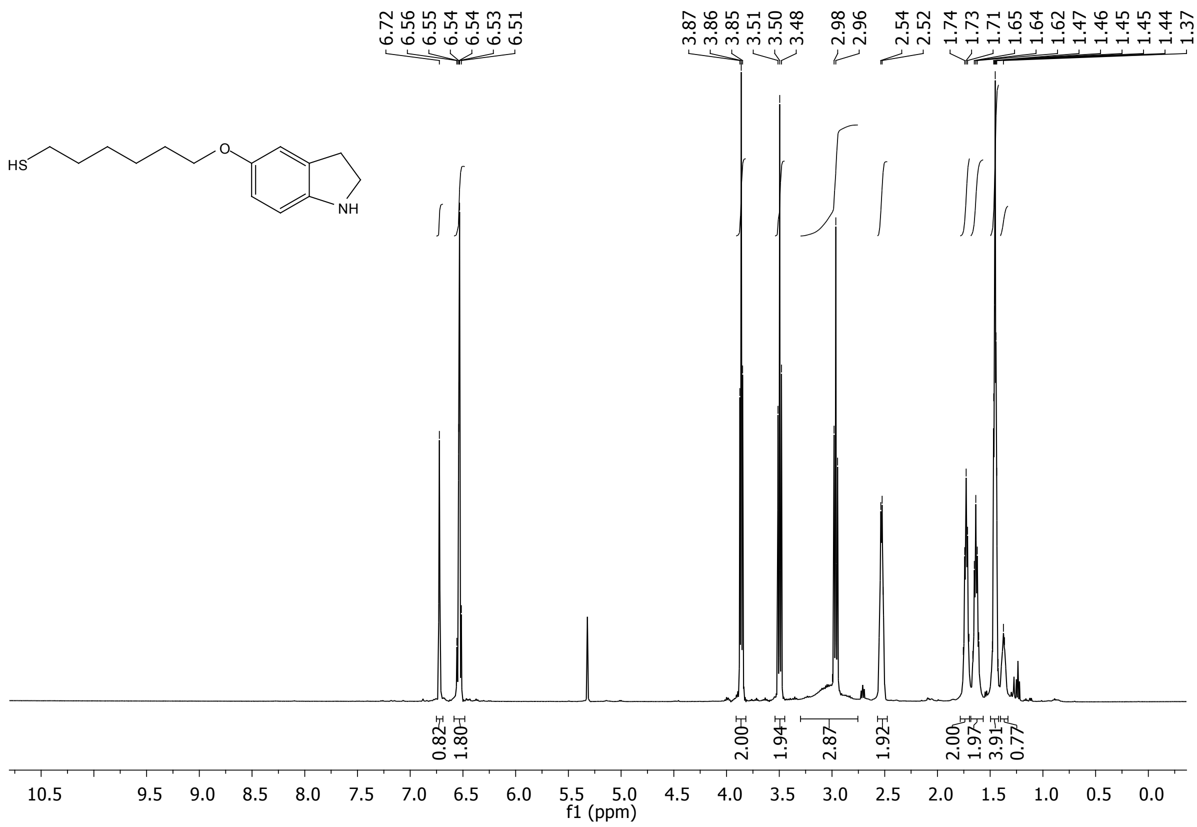

Figure S28. ^{1}H NMR spectrum of **5** in CD_2Cl_2 (500 MHz).

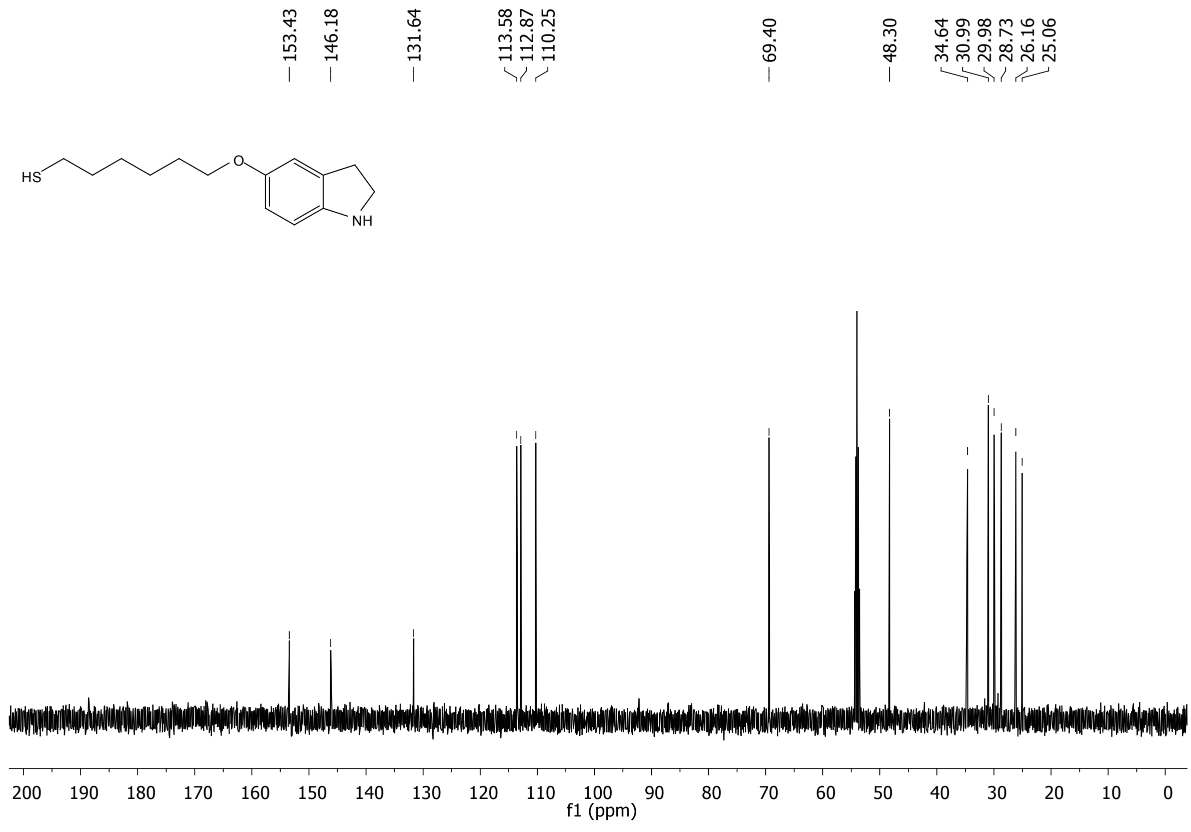

Figure S29. ^{13}C NMR spectrum of **5** in CD_2Cl_2 (500 MHz).

References

1. J. R. Hemmer, Z. A. Page, K. D. Clark, F. Stricker, N. D. Dolinski, C. J. Hawker, J. R. de Alaniz, *J. Am. Chem. Soc.* **2018**, *140*, 10425–10429.

2. Zhao, H.; Wang, D.; Fan, Y.; Ren, M.; Dong, S.; Zheng, Y. Surface with Reversible Green-Light-Switched Wettability by Donor−Acceptor Stenhouse Adducts. *Langmuir* **2018**, *34*, 15537−15543.

3. Singh, S.; Mai, P.; Borowiec, J.; Zhang, Y., Lei, Y.; Schober A. Donor–Acceptor Stenhouse Adduct-Grafted Polycarbonate Surfaces: Selectivity of the Reaction for Secondary Amine on Surface. *R. Soc. Open Sci.* **2018**, *5*, 180207.

4. Hemmer, J. R.; Poelma, S. O.; Treat, N.; Page, Z. A.; Dolinski, N. D.; Diaz, Y. J.; Tomlinson, W.; Clark, K. D.; Hooper, J. P.; Hawker, C.; Read de Alaniz, J. Tunable Visible and Near Infrared Photoswitches. *J. Am. Chem. Soc.* **2016**, *138*, 13960−13966.

5. Di Donato, M.; Lerch, M. M.; Lapini, A.; Laurent, A. D.; Iagatti, A.; Bussotti, L.; Ihrig, S. P.; Medved', M.; Jacquemin, D.; Szymanski, W.; Buma, W. J.; Foggi, P.; Feringa, B. L. Shedding Light on the Photoisomerization Pathway of Donor−Acceptor Stenhouse Adducts. *J. Am. Chem. Soc.* **2017**, *139*, 15596−15599.

6. Alves, J.; Wiedbrauk, S.; Gräfe, D.; Walden, S. L.; Blinco, J. P.; Barner-Kowollik, C. It's a Trap: Thiol-Michael Chemistry on a DASA Photoswitch. *Chem. Eur. J.* **2020**, *26*, 809–813.

7. Ahrens, J.; Bian, T.; Vexler, T.; Klajn, R. Irreversible Bleaching of Donor–Acceptor Stenhouse Adducts on the Surfaces of Magnetite Nanoparticles. *ChemPhotoChem* **2017**, *1*, 230–236.

8. Mohai, M. XPS MultiQuant: Multimodel XPS Quantification Software. *Surf. Interface Anal.* **2004**, *36*, 828–832. http://aki.ttk.mta.hu/XMQpages/XMQhome.htm (last access: January 2017).

9. Evans, S.; Pritchard, R. G.; Thomas, J. M. Relative Differential Subshell Photoionization Cross-sections (Mg Kα) from Lithium to Uranium. *J. Electron Spectrosc. Relat. Phenom.* **1978**, *14*, 341–358.

10. Reilman, R. F.; Msezane, A.; Manson, S. T. Relative Intensities in Photoelectron Spectroscopy of Atoms and Molecules. *J. Electron Spectrosc. Relat. Phenom.* **1976**, *8*, 389–394.

11. Kunfi, A.; Vlocskó, R. B.; Keresztes, Z.; Mohai, M.; Bertóti, I.; Ábrahám, Á.; Kiss, É.; London, G. Photoswitchable Macroscopic Solid Surfaces Based On Azobenzene-Functionalized Polydopamine/Gold Nanoparticle Composite Materials: Formation, Isomerization and Ligand Exchange. *ChemPlusChem* **2020**, *85*, 797–805.

12. *Gaussian 09*, Revision E.01, Frisch, M. J.; Trucks, G. W.; Schlegel, H. B.; Scuseria, G. E.; Robb, M. A.; Cheeseman, J. R.; Scalmani, G.; Barone, V.; Petersson, G. A.; Nakatsuji, H.; Li, X.; Caricato, M.; Marenich, A. V.; Bloino, J.; Janesko, B. G.; Gomperts, R.; Mennucci, B.; Hratchian, H. P.; Ortiz, J. V.; Izmaylov, A. F.;

Sonnenberg, J. L.; Williams-Young, D.; Ding, F.; Lipparini, F.; Egidi, F.; Goings, J.; Peng, B.; Petrone, A.; Henderson, T.; Ranasinghe, D.; Zakrzewski, V. G.; Gao, J.; Rega, N.; Zheng, G.; Liang, W.; Hada, M.; Ehara, M.; Toyota, K.; Fukuda, R.; Hasegawa, J.; Ishida, M.; Nakajima, T.; Honda, Y.; Kitao, O.; Nakai, H.; Vreven, T.; Throssell, K.; Montgomery, J. A., Jr.; Peralta, J. E.; Ogliaro, F.; Bearpark, M. J.; Heyd, J. J.; Brothers, E. N.; Kudin, K. N.; Staroverov, V. N.; Keith, T. A.; Kobayashi, R.; Normand, J.; Raghavachari, K.; Rendell, A. P.; Burant, J. C.; Iyengar, S. S.; Tomasi, J.; Cossi, M.; Millam, J. M.; Klene, M.; Adamo, C.; Cammi, R.; Ochterski, J. W.; Martin, R. L.; Morokuma, K.; Farkas, O.; Foresman, J. B.; Fox, D. J. Gaussian, Inc., Wallingford CT, **2013**.

13. Stephens, P. J.; Devlin, F. J.; Chabalowski, C. F.; Frisch, M. J. Ab Initio Calculation of Vibrational Absorption and Circular Dichroism Spectra Using Density Functional Force Fields. *J. Phys. Chem.* **1994**, *98*, 11623–11627.

14. Hehre, W.J.; Ditchfireld, R.; Pople, J. A. Self–Consistent Molecular Orbital Methods. XII. Further Extensions of Gaussian–Type Basis Sets for Use in Molecular Orbital Studies of Organic Molecules. *J. Chem. Phys.* **1972**, *56*, 2257–2261.

15. Krishnan, R.; Binkley, K. S.; Seeger, R.; Pople, J. A. Self-Consistent Molecular Orbital Methods. XX. A Basis Set for Correlated Wave Functions. *J. Chem. Phys.* **1980**, *72*, 650–654.
